# Hydricity of 3d Transition Metal Complexes from Density Functional Theory: A Benchmarking Study

**DOI:** 10.3390/molecules26134072

**Published:** 2021-07-03

**Authors:** Alister S. Goodfellow, Michael Bühl

**Affiliations:** School of Chemistry, University of St Andrews, St Andrews KY16 9ST, UK; ag266@st-andrews.ac.uk

**Keywords:** DFT, 3d metal complex, benchmark, hydricity

## Abstract

A range of modern density functional theory (DFT) functionals have been benchmarked against experimentally determined metal hydride bond strengths for three first-row TM hydride complexes. Geometries were found to be produced sufficiently accurately with RI-BP86-D3(PCM)/def2-SVP and further single-point calculations with PBE0-D3(PCM)/def2-TZVP were found to reproduce the experimental hydricity accurately, with a mean absolute deviation of 1.4 kcal/mol for the complexes studied.

## 1. Introduction

At the forefront of modern chemistry is sustainability, with a drive towards ‘greener’ processes and development. Catalysis has always been a tool used to reduce energetic costs and to promote specific reactions, improving selectivity and the efficacy of various transformations. Traditionally, homogeneous catalysts have been based upon expensive and unsustainable metals such as platinum, palladium and rhodium [1] and while these 4d and 5d transition metals (TMs) have been very successful in this application, their future use is limited by concerns over sustainability and price. The scarcity of the metals has resulted in a high economic and social cost in their extraction. The metals themselves are also toxic, which may lead to issues of contamination in extraction, chemical transformations, or in the application of these catalysts in industrial processes. To alleviate these issues, development has moved towards the use of 3d TMs, which are largely more abundant, less toxic and more sustainable [2,3,4,5].

**Scheme 1:**
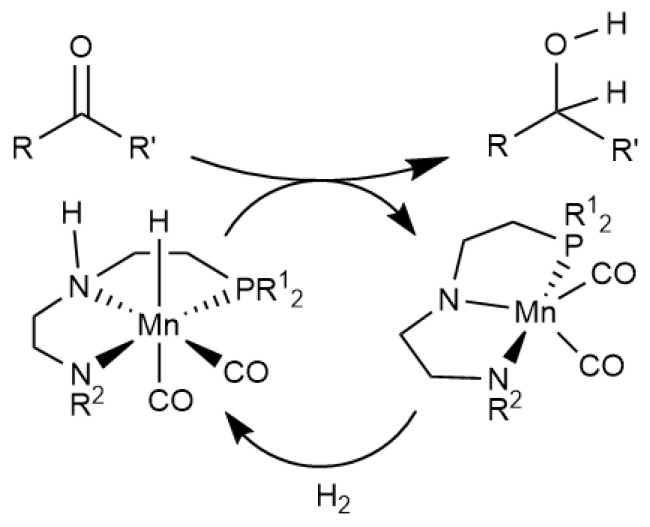
A representation of the catalytic application of Mn-based systems from the group of Beller [9].

First-row TM catalysts have undergone huge development in the past few years and are being optimised to ultimately become competitive against their unsustainable heavy metal congeners. Iron and manganese are attractive due to their low toxicity and high abundance, and work on catalytic hydrogenation reactions using these metals is very topical (see Scheme 1). For a review on the development of manganese based pincer catalysts, see Garbe et al. [6].

Density functional theory is a powerful tool for the elucidation of reaction mechanisms and for the rational design of these catalytic systems. To accurately study such systems, the DFT functional used must be suitable for the system, accurately predicting bond energies that are crucial to the functionality of the molecule. 3d TMs are notoriously tricky to study with DFT, especially bare TMs where the electronic structure is not always predicted correctly [7,8]. For investigation in the field of homogeneous catalysis, especially in hydrogenation reactions as shown in Scheme 1, the metal hydride bond will be of prime importance for the catalytic activity, and the functional used must be accurate in the description of these bond strengths.

A number of papers have used DFT computations to examine the reactivity of these catalytic systems and a variety of methodology has been employed (Table 1). The choice of methodology may be based upon previous work in related catalysis, perhaps 4d metal systems, or simply by using methods that have already been applied in the literature. For example, early computations from the Beller group used B3PW91, based upon the performance of this hybrid functional in previous work (including benchmarking studies) across 3d, 4d and 5d transition metal complexes though more recent work from this group has used the hybrid PBE0 functional [9,10,11,12,13,14,15].

An alternate approach was adopted by Ge et al. [16], where the methodology was selected based upon the testing of a range of functionals for a multi-step transformation. M06 was selected for their work as the computed barrier heights were found to be closest to the average value across the functionals tested. A benchmarking study was performed by Gamez et al. [17] on a number of high-spin Mn-based complexes using a range of different functionals. It was found that pure functionals (i.e., non-hybrid) favoured low-spin forms of these Mn complexes, with weak-field nitrogen and oxygen based ligands, experimentally known to be high-spin complexes. B3LYP was found to produce poor geometries and PBE0 was recommended for use on these systems. Dispersion corrections were also found to be of importance in optimisation. The accurate description of spin states is important in the study of 3d metal complexes, where the energetic splitting of metal-based 3d orbitals may be small and multi-state reactivity may be possible.

We now present a validation study on the performance of these and other functionals for the calculation of hydricity for a few select complexes. Hydricity is the heterolytic bond dissociation energy of a metal hydride to a metal cation and hydride (Equation (1)), predicted to be an important property for the study of hydrogenation catalysis.

(1) $$MH⇌M^{+}+H^{-}\;\Delta G_{H-}^{\circ }$$

A sizeable amount of $\Delta G_{H^{-}}^{\circ }$ data is available from experiment [39], the ultimate benchmark. Arguably, this quantity is key in determining the ease of elementary steps such as transfer of the hydride to an electrophilic substrate (*cf.* upper arrow in Scheme 1). As it turns out, there is a notable variability in the performance of different functionals in their ability to reproduce these hydricity values, and some clear recommendations for functionals to choose can be made.

## 2. Methodology

### 2.1. Computational Details

Geometry optimisations were performed primarily at the GGA level of DFT using the BP86 functional, employing a variety of basis sets from the redefinitions of the Ahlrichs family of bases [40,41,42,43]. Structures obtained by X-ray crystallography for closely related systems were used as starting points in geometry optimisations [44,45,46]. This is akin to the methodology of Neale [47] used in the investigation of Fe(II) and Co(III) catalysis and of Bühl and Kabrede [48] in the benchmarking of geometries for a wide range of first-row transition metal complexes. Def2-TZVP was used for the investigations of proton and metal cation solvation and throughout further benchmarking, a variety of basis from this family were used; def2-SVP, with def2-TZVP describing the metal atom of Fe or Co, and the addition of a diffuse function on the hydridic hydrogen, taking ⅓ of the exponent from the diffuse ‘s’ function in the def2-TZVP basis for hydrogen. Dispersion corrections were included throughout, predominantly with the D3 empirical correction from Grimme [49], including Becke-Johnson dampening [50]. Implicit solvation was also included using the Polarisable Continuum Model with the integral equation formalism (IEF-PCM, herein referred to as PCM) employing the parameters of acetonitrile (ϵ=35.688). The explicit treatment of a small number of individual solvent molecules acting as ligands was also considered (Section 3.1). The nature of the minima obtained were verified by computation of harmonic frequencies at the same level of theory. Single-point energies were obtained using a number of functionals from a range of levels on Jacob’s ladder [51] using the def2-TZVP basis, with an ultrafine integration grid (99 radial shells with 590 angular points per shell). The impact of using a larger, quadruple-ζ basis, def2-QZVP, was also explored. The functionals considered were; B3LYP [52,53,54], B3PW91 [52,55,56,57], BLYP [53,54,58], BP86 [58,59], CAM-B3LYP [60], M06 [61], M06-2X [61], M06-HF [62,63], M06-L [64], M11 [65], M11-L [66], MN12-L [67], MN15 [68], MN15-L [69], PBE [55,70], PBE0 [71], PBE0-⅓ [72], revPBE [73], revPBE0 [73], TPSS [74], TPSSh [74,75], ωB97X-D [76]. Dispersion was included for all single-point calculations as mentioned earlier, except for the Minnesota family of functionals, which are parametrised to account for dispersion, and the ωB97X-D functional includes the D2 correctional term from Grimme [77]. Thermochemistry was evaluated at 468 atm (to mimic bulk acetonitrile, see Section 1 in the ESI for details) and 298.15 K using thermodynamic corrections obtained at the level of the geometry optimisation, combined with single-point energetics. Density fitting, invoking the RI approximation with the Gaussian keyword ‘auto’, was used where possible for pure functionals. Calculations were performed primarily using Gaussian 09, D.01 [78], with some single-points evaluated using Gaussian 16, C.01 [79] where the functional was unavailable in Gaussian 09. Some geometries were also evaluated using the more recent optimiser of Gaussian 16 to remove spurious imaginary frequencies that could not be removed with Gaussian 09. Where Gaussian 16 was used, defaults from the older Gaussian 09 were employed to ensure that there was no change in implementation across different versions (see Table S5 in the ESI).

### 2.2. Calculation of Hydricity

To determine a functional suitable for the study of 3d TM catalysts (for example, see Scheme 1), we have chosen to benchmark three 3d TM hydride complexes, for which there are experimentally determined hydricity values ($\Delta G_{H^{-}}$, Table 2) [39]. Direct evaluation of hydricity according to Equation (1) is challenging computationally. In this work we use a thermodynamic cycle with a protonation as key step, where the ionic stoichiometry is maintained across the reaction (i.e. charge separation is avoided, Equation (2)). This has been done to ensure that there is no artefact arising from an imbalance of ionic species, as their large stabilisation in a polar solvent is difficult to compute accurately using methods employing implicit solvation, such as PCM (as would be the case by directly modelling Equation (1)). By combination with H_2_ heterolysis obtained from experiment [39] (Equation (3)), the thermochemical cycle may be completed to obtain the thermodynamic hydricity.

(2) $$\begin{array}{cc} \begin{array}{c} MH+H^{+}⇌M^{+}+H_{2} \end{array} & \Delta G_{1}^{\circ } \end{array}$$

(3) $$\begin{array}{cc} \begin{array}{c} H_{2}⇌H^{+}+H^{-} \end{array} & \Delta G_{H_{2}}^{\circ }=76.0\;\mathrm{kcal/mol}\;[39] \end{array}$$

With limited availability of Mn based data, neutral Co and Fe hydride complexes have been chosen from data by Wiedner et al. [39] as representatives of 3d metal hydride systems. Benchmarking studies have previously been performed on 3d TM diatomics [83], but the applicability of these studies to such larger complexes remains unclear.

### 2.3. Acetonitrile Proton Clusters

The proton involved in Equation (2) will be modelled through a complex with discrete solvent molecules. Experimentally, acetonitrile had been used as a solvent and it was observed by Himmel et al. [84] that, in acetonitrile, proton-solvent clusters were present in the form of extended hydrogen bonded arrays, [H(NCMe)_*n*_]^+^, with values of *n* up to 8. For the purpose of this study, we determined that the proton-solvent cluster was suitably described with two explicit solvent molecules (n=2) forming an inner solvent shell and approximation of the outer solvent shell with implicit solvation using a polarisable continuum model (further details are available in Section 2 of the ESI).

## 3. Results and Discussion

### 3.1. Explicit Treatment of Solvent Molecules

Implicit solvation is used as a way to approximate the solvation of gas phase molecules, with continuum models used to describe a solvation shell around a molecule as a dielectric; however, this does not describe specific interactions, where a solvent molecule may act as a ligand to stabilise an ionic species (Equation (4)). In such cases, the explicit treatment of a solvent molecule is required to describe the role as a ligand in the coordination sphere and as such, the reaction shown in Equation (2) may be better represented by the reaction in Equation (5), where both cations are solvated by explicit solvent molecules.

(4) $$\begin{array}{cc} \begin{array}{c} {\left[M(NCMe\right]}^{+}⇌M^{+}+MeCN \end{array} & \Delta G_{BDE}^{\circ } \end{array}$$

(5) $$\begin{array}{cc} \begin{array}{c} MH+{\left[H(NCMe{)}_{2}\right]}^{+}⇌{\left[M(NCMe)\right]}^{+}+MeCN+H_{2} \end{array} & \Delta G_{2}^{\circ } \end{array}$$

If a solvent molecule is coordinated to the metal centre after hydride dissociation, according to Equation (1), this binding energy is implicitly included in the experimental determination of thermodynamic hydricity by Wiedner et al. [39] (Equation (2)). Across the three complexes used (Table 2), both the sterics and accessibility of the solvent to the coordination shell are significantly different. In cationic metal complexes, where a vacant coordination site is exposed and accessible, a solvent molecule may strongly bind to the metal centre, stabilising the complex to a higher degree than would be predicted with only the inclusion of a dielectric model. Here, the bond dissociation energy of the ligand (Equation (4)) becomes important and a solvent molecule should be treated explicitly if there is a strong interaction between the cationic metal complex and solvent molecule.

The Fe cationic complex has a low steric bulk and vacant and accessible coordination sphere, [FeCp(CO)_2_]^+^. In contrast, the two Co complexes have large ligands attached to each metal centre and are far more sterically crowded (see 3D surface plots in Table 2). We found that, for the most accurate computed hydricity values, the Fe cationic species must be treated with an explicitly bound solvent molecule, whereas the Co complexes do not require this (Equations (6)–(8)). While there is an element of functional dependence, this methodology has been tested and was found to produce the lowest mean absolute errors (MAEs) in the calculation of hydricity when compared to the values obtained by Wiedner et al. [39] (further details are available in Section 3 of the ESI).

(6) $$\begin{array}{cc} \begin{array}{c} FeCp(CO{)}_{2}H+[H(NCMe{)}_{2}{]}^{+} \end{array} & ⇌[FeCp(CO{)}_{2}(NCMe){]}^{+}+MeCN+H_{2} \end{array}$$

Co(P_4_N_2_)H + [H(NCMe)_2_]^+^ ⇌ [Co(P_4_N_2_)]^+^ + 2MeCN + H_2_ (7)

Co(dppe)_2_H + [H(NCMe)_2_]^+^ ⇌ [Co(dppe)_2_]^+^ + 2MeCN + H_2_ (8)

### 3.2. Choice of Single-Point Functional

To quantitatively predict the thermodynamic hydricity of a complex, the ability of the functional used to produce accurate energies is of prime importance. Single-point energy calculations have been performed with a range of functionals, on geometry optimised structures at the level of RI-BP86-D3(PCM_MeCN_), with a large triple-ζ basis, def2-TZVP, from the group of Ahlrichs [40,41,42,43].

**Figure 1:**
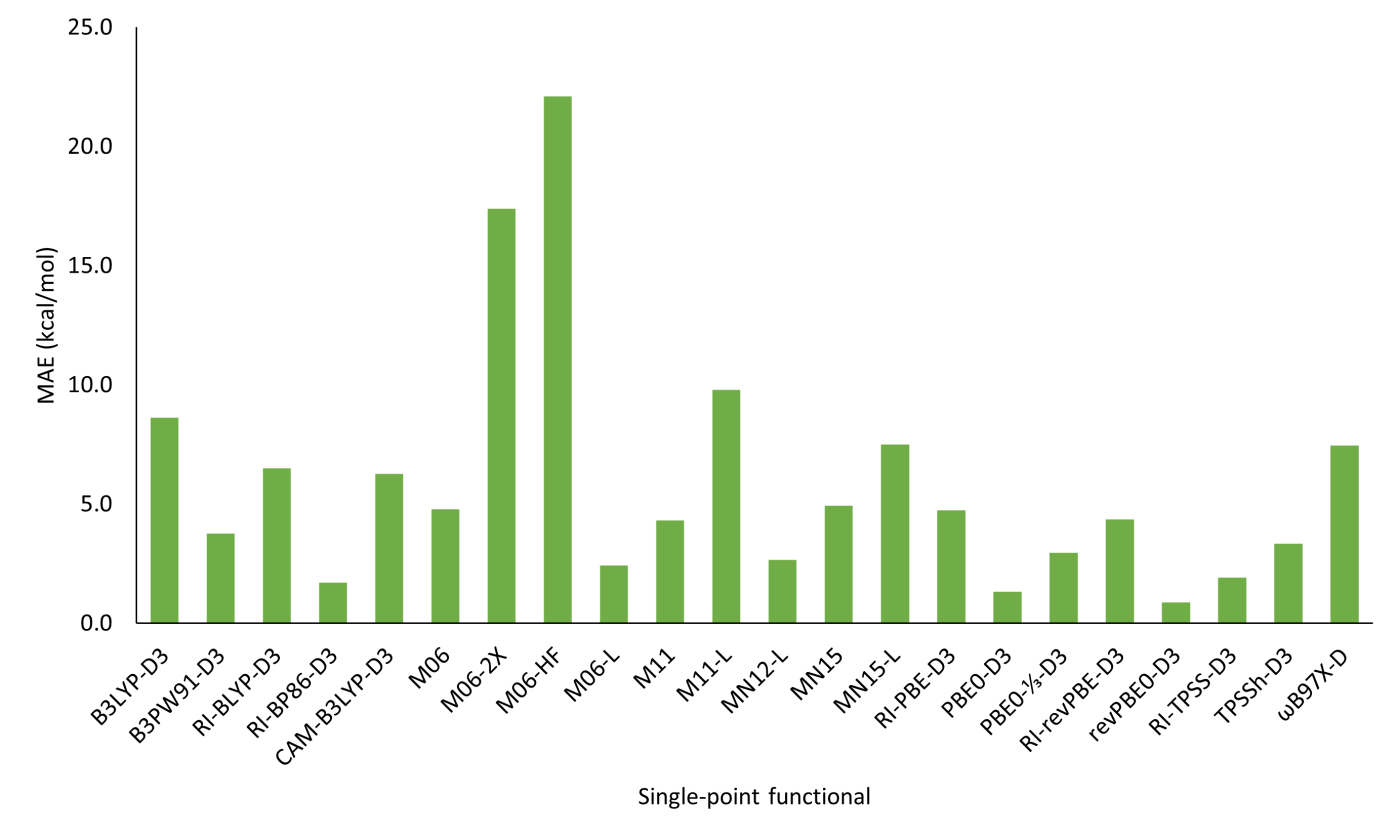
MAE in the calculation of hydricity across each of the three complexes with a range of single-point functionals. Performed with implicit solvation (PCM_MeCN_) and a def2-TZVP basis on optimised geometries from RI-BP86-D3(PCM_MeCN_)/def2-TZVP.

The functional used in the single-point calculation was found to have a significant impact upon the ability to quantitatively reproduce thermodynamic hydricity values obtained from experiment. Errors in the form of mean absolute error, have been calculated for each functional across the three complexes (Equation (9) and Figure 1).

(9) $$MAE=\frac{1}{n}\Sigma \left|E_{calc}-E_{exp}\right|$$

Clearly, the Minnesota functionals of M06-2X and M06-HF, both highly parameterised for main group chemistry, are poor in their description of these 3d TM complexes. The lowest MAEs were obtained from RI-BP86, PBE0 and revPBE0 (1.7, 1.3 and 0.9 kcal/mol). The percentage of HF exchange has been shown to be important by Moltved et al. [83] and this effect can be seen in the comparison of PBE0 with PBE0-⅓, with 25% and 33% HF exchange, respectively. Both forms of the hybrid PBE functional performed well in terms of overall performance, yet PBE0-⅓ had over twice the MAE of PBE0 (1.3 and 2.9 kcal/mol, respectively).

Based purely upon the MAEs obtained here, revPBE0 would be the functional of choice; however, we recommend to use PBE0 (based upon an improved performance over revPBE0 on geometries produced with the smaller def2-SVP basis, MAEs of 1.4 and 1.8 kcal/mol, see ESI Figure S7 and Section 3.3.2), which has been shown to perform well in many previous studies of TM systems [17,85] and in the differentiation of spin state energetics [17].

### 3.3. Validation of Methodology

We have carefully tested the robustness of the benchmark data summarised in Figure 1 against other choices of methodological details. These comprise:

#### 3.3.1. Choice of Basis Set for Single-Point Energies

Single-point calculations in this work were performed using the def2-TZVP basis from Ahlrichs. The impact of a larger, quadruple-ζ basis, def2-QZVP, was examined on the smallest Fe system for a selection of single-points (Figure S4 in the ESI).

For the additional expense of using a quadruple-ζ basis in the description of the system, there was no significant improvement to be found. Def2-TZVP has been shown to be of sufficient accuracy for single-point calculations and a greater emphasis is placed upon the choice of functional for these 3d TM hydride complexes.

#### 3.3.2. Level of Geometry Optimisation

RI-BP86 was initially chosen for use in geometry optimisation as a ‘tried and tested’ method [48]. From comparison of BP86 with PBE0, M06 and M06-L, it was found that there was no benefit to the use of the more expensive hybrid, meta-hybrid or meta-GGA functional over the pure functional in the geometry optimisation (MAEs of 1.4, 4.8, 4.5 and 4.3 kcal/mol, respectively, Figure S5 in the ESI).

Furthermore, under consideration was the use of a different basis in the optimisation. In general, use of the largest, triple-ζ, def2-TZVP basis set was found to perform best, with a reduction in MAE compared to the smaller double-ζ, def2-SVP basis. Though, we did find that the description of the hydridic hydrogen with an additional diffuse function, or of the metal centre with a larger basis, was not always found to improve the MAE (see Figure S7 in the ESI). While the triple-ζ basis was found to offer a slight improvement, the associated expense of using this larger basis means that this is impractical for the fractional improvement and the smaller, double-ζ basis is deemed sufficient for geometry optimisation of these systems (MAEs of 1.3 and 1.4 kcal/mol, respectively, from single-points of PBE0-D3(PCM_MeCN_)/def2-TZVP, but with CPU times for the frequency calculation, at the level of geometry optimisation, for Co(dppe)_2_H of approximately 12.5 days compared to 2 days for def2-TZVP and def2-SVP basis sets, respectively). Additionally, there was little change in the optimised geometry between the use of def2-SVP and def2-TZVP, with central metal ligand bond distances reproduced to within 0.006 Å (see Table S3 in the ESI for a comparison to X-Ray crystal structures from Ciancanelli et al. [81] and Ariyaratne et al. [86]).

Theoretically, for the most accurate description of the system, both dispersion and solvation should be considered and indeed we found that the inclusion of both minimised the MAEs in the calculation of hydricity across the three complexes (Figure S8 in the ESI).

#### 3.3.3. Choice of Solvation Model

The choice of solvent model has been examined at the level of single-point calculations and there was no significant variation of the hydricity values obtained by using three different solvation models; IEF-PCM, C-PCM and SMD (Table S4 in the ESI).

## 4. Conclusions

We have accurately reproduced experimentally measured values of hydricity for three 3d TM complexes. While a mixture of functionals have been used in the literature for studies on 3d metal homogeneous catalysis, we propose a methodology that has been shown to accurately reproduce a key M−H bond strength, central to the reactivity of these compounds.

While low on the Jacob’s ladder of functionals, the pure GGA, BP86, has been shown to produce accurate energetics for the hydricity of 3d TM hydrides. The hybrid functional, PBE0 has also been shown to perform well and is recommended for energy calculations over BP86 due to the improved ability to more reliably differentiate between spin states of 3d TM complexes [17]. The lowest mean absolute errors were found with the inclusion of both dispersion corrections and implicit solvation.

A double-ζ basis, def2-SVP, was used in geometry optimisation with the RI-BP86-D3(PCM) and led to a MAE of 1.4 kcal/mol after evaluation of subsequent single-point at the level of PBE0-D3(PCM)/def2-TZVP. A larger triple-ζ basis, def2-TZVP, used at the stage of the geometry optimisation led to a lower MAE of 1.3 kcal/mol which, was not shown to offer any significant improvement for the additional cost.

For a balance between accuracy and expense, we recommend the methodology of PBE0-D3(PCM)/def2-TZVP//RI-BP86-D3(PCM)/def2-SVP for use on systems involving 3d TM hydride complexes.

## Figures and Tables

**Table 1:** A summary of the literature methodology used for DFT calculations on 3d metal catalytic systems.

System	Methodology ^a^	Dispersion ^b,c^	Solvent Model ^c^	References
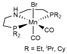	B3PW91/TZVP			[9,10,15]
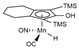	M06/6-31++G(d,p)	M	PCM	[16]
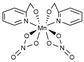	PBE0/def2-TZVP			[17]
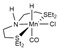	ωB97X-D/ECP (Mn)/6-31++G(d,p)	GD2	PCM	[18]
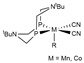	M06/ECP (Mn,Co)/6-31(+)G(d,p)	M	PCM	[19]
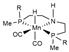	ωB97X-D/ECP (Mn)/cc-PVTZ	GD2	PCM	[20]
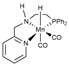	M06/TZVP// ωB97X-D/TZVP (Mn)/SVP	M sp GD2 opt	PCM	[21]
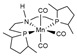	B3PW91/TZVP			[22]
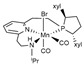	ωB97X-D/ECP (Mn)/cc-pVDZ	GD2	PCM	[23]
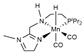	M06L/def2-TZVP// B3LYP/def2-SVP	M sp	SMD sp	[24]
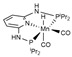	PBE0/ECP (Mn)/6-31G(d,p)		PCM	[25]
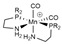	PBE0/6-311+G(d,p)// PBE0/6-31G(d,p)			[26]
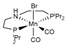	DSD-PBE96-NL/def2-QZVP// PBE0-D3/def2-TZVP	GD3BJ opt		[27]
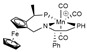	M06/6-311++G(d,p)// M06/ECP (Mn)/6-31G(d)	M	SMD sp	[28]
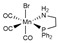	PBE0/6-311+G(d)// PBE0/6-31G(d)		PCM sp	[29]
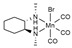	PBE0/def2-QZVPP// PBE0-D3/def2-TZVP	GD3BJ opt	SMD opt	[30]
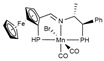	PBE0/ECP (Mn)/6-31G(d,p)			[31]
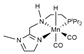	B3LYP/ECP (Mn)/6-311++G(d,p)		CPCM	[32]
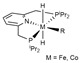	ωB97X/6-31++G(d,p)		PCM	[33]
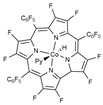	BP86/6-311+G(d)// BP86/6-311G(d) (Co)/6-31G(d)		PCM sp	[34]
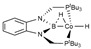	B3PW91/ECP (Co)/6-31++G(d,p)// B3PW91/ECP (Co)/6-31++G(d,p) (B,N,P)/6-31G(d)	GD sp GD opt	CPCM sp	[35]
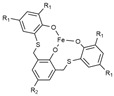	M06/TZVP// BP86/ECP (Fe)/SVP	M sp	PCM sp	[36]
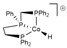	B3PW91/def2-TZVP// B3PW91/TZVP	GD3BJ sp	SMD sp	[37]
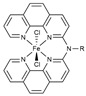	B3LYP*/ECP (Fe)/6-311+G(2df,2p)// B3LYP/ECP (Fe)/6-31G(d,p)	GD3BJ sp GD3BJ opt	SMD sp	[38]

^a^ For simplicity, the use of any pseudopotential has been indicated with ECP. ^b^ M denotes that there is implicit dispersion included, due to the parametrisation of the Minnesota family of functionals. GD is used to indicate the inclusion of Grimme’s empirical dispersion correction, with BJ denoting the use of Becke-Johnson dampening. ^c^ ‘sp’ and ‘opt’ denote the use of dispersion corrections or solvation models in either the single-point or geometry optimisation calculation, respectively.

**Table 2:** Three 3d transition metal complexes with experimentally determined thermodynamic hydricity values $\left(\Delta G_{H^{-}}\right)$.

Complex	FeCp(CO)_2_H	Co(dppe)_2_H	Co(P_4_N_2_)H
$\Delta G_{\begin{array}{c} H^{-} \end{array}}$ (kcal/mol)	61.7 [39,80]	49.9 [81,82]	31.8 [82]
	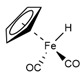	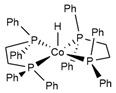	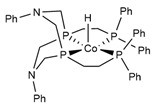
	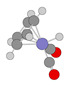	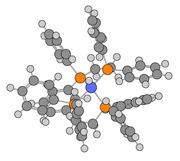	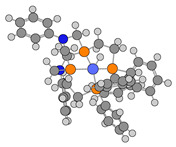
Positively charged cationic complex fragment after hydride dissociation ^a^	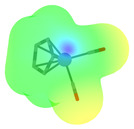	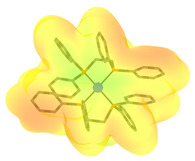	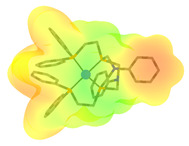

^a^ Hydrogen atoms have been removed for clarity. The viewing angle is looking towards the vacant site on the metal centre where the hydridic hydrogen atom was residing previously, emphasising the accessibility of the now vacant site by a solvent molecule. Electron density (isodensity value of 0.001 a.u.) mapped with electrostatic potential [colour-coded between 0.00 a.u. (red) and +0.35 a.u. (blue)].

## Data Availability

The research data supporting this publication can be accessed at https://doi.org/10.17630/7cf7b7e9-3edc-4ad4-b016-3b9b5548f9ac (accessed on 2 July 2021).

## Supplementary Materials

The following are available online at, additional computational details and graphical and tabular material.
