# Supplementary material for: Hydricity of 3d Transition Metal Complexes from Density Functional Theory: A Benchmarking Study
Source: Molecules. 2021 Jul 3;26(13):4072. doi: 10.3390/molecules26134072 (PMC8271472; doi:10.3390/molecules26134072)
Supplement: Supplementary file 1 [file molecules-26-04072-s001.zip › molecules-1267692-supplementary.pdf]

# Hydricity Of 3d Transition Metal Complexes From Density Functional Theory: A Benchmarking Study – Electronic Supplementary Information

Alister S. Goodfellow and Michael Bühl

## Contents

**1 Derivation of Martin, Hay, Pratt Entropic Correction** **2**

**2 Acetonitrile Proton Clusters** **3**

**3 Binding of Solvent to Metal Cation** **4**

**4 Methodological Considerations** **6**
4.1 Choice of Single-Point Basis . . . . . 6
4.2 Description of Geometries . . . . . 7
4.2.1 Choice of Functional . . . . . 7
4.2.2 The Effect of Basis Set in Geometry Optimisation . . . . . 8
4.2.3 Optimisation Conditions . . . . . 8
4.2.4 Comparison of X-Ray and Optimised Structures . . . . . 9
4.3 Solvation Model . . . . . 10
4.4 Use of Different Gaussian Versions . . . . . 10
4.5 Hydride Binding Energy . . . . . 10

**References** **11**

**5 Cartesian Coordinates of Optimised Structures** **12**

# 1 Derivation of Martin, Hay, Pratt Entropic Correction

In solution, quantitative prediction of reaction entropy is a challenge due to a restriction in degrees of freedom of the solute imposed by the solvent [1]. As such, there is often an overestimation of the entropy contribution to the Gibbs free energy in solution. Martin, Hay and Pratt [2] introduced an empirical correction for this overestimation, artificially raising the pressure of water to 1354 atm, where the density of the ideal gas phase is equal to the liquid phase. This corresponds to a correctional term of (n − m) × 4.3 kcal/mol for the reaction of mR $\rightleftharpoons$ nP. This has been used in previous studies on catalytic systems by this group [3–8]. The derivation of the correctional term for the solvent acetonitrile is shown;

$$p = \frac{\rho RT}{M} = 468.45 \text{ atm}, \quad \text{(S1)}$$

$$S_{MHP} = RT\,ln\frac{p}{p^{\circ}} = 3.643 \text{ kcal/mol per particle}, \quad \text{(S2)}$$

where $p$ = MeCN pressure, $\rho$ = density of MeCN (0.786 g/cm$^3$), $M$ = molar mass of MeCN (41.05 g/mol), T = 298.15 K, $p^{\circ}$ = 1 atm, $R$ = ideal gas constant (8.314 J/(K mol)) and $S_{MHP}$ = Martin, Hay, Pratt entropic correctional term.

## 2 Acetonitrile Proton Clusters

It was previously shown by Himmel *et al.* [9] that in acetonitrile, proton solvent clusters were present in the form of extended hydrogen bonded arrays, $[H(NCMe)_n]^+$, with values of $n$ up to 8 (Equation S3). To determine the relative stability of such proton clusters, the free energy change associated with the reaction in Equation S4, was evaluated (Table S1).

$$[H(NCMe)_n]^+ \rightleftharpoons H^+ + n\text{MeCN} \qquad \Delta G^\circ \tag{S3}$$

$$[H(NCMe)_n]^+ \rightleftharpoons [H(NCMe)]^+ + (n-1)\text{MeCN} \qquad \Delta G^\circ \tag{S4}$$

**Table S1** $\Delta G^\circ$ for the reaction in Equation S4. Computations were performed at the level of RI-BP86-D3(PCM$_{\text{MeCN}}$)/def2-TZVP with values in kcal/mol.

| ***n*** | *1* | *2* | *3* | *4* | *5* | *6* | *7* |
|---|---|---|---|---|---|---|---|
| $\Delta G^\circ$ | 0.0 | 13.5 | 9.8 | 9.0 | 8.5 | 6.4 | 6.5 |

From the transformation shown in Equation S4, the highest value of $\Delta G^\circ$ is indicative of the most stable acetonitrile proton cluster relative to proton 'cluster' containing a single solvent molecule. Based on this methodology, the cluster involving two acetonitrile solvent molecules is predicted to be the most stable.

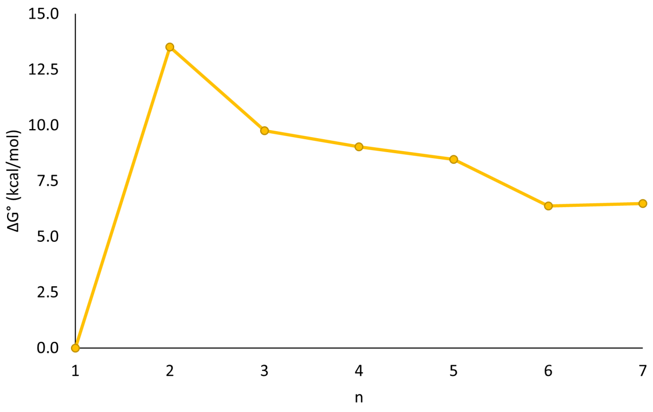


**Figure S1** Graph of $\Delta G^\circ$ against $n$ from the reaction shown in Equation S4. Computations were performed at the level of RI-BP86-D3(PCM$_{\text{MeCN}}$)/def2-TZVP and values are presented with units of kcal/mol.

# 3 Binding of Solvent to Metal Cation

The free energy change of the reaction in Equation S5 has been calculated with the range of functionals that have been considered in this benchmarking study and the free energy of binding ($\Delta G^\circ$ + (m - n) $\times$ $S_{MHP}$) has been plotted for each functional and metal complex (Figure S2).

$$[\mathrm{ML(NCMe)}]^+ \rightleftharpoons [\mathrm{ML}]^+ + \mathrm{MeCN} \qquad \Delta G^\circ_{BDE} \tag{S5}$$

As can be seen from the graph in Figure S2, the Fe complex has been calculated to have strong binding affinity to an explicit solvent molecule due to the low steric bulk and vacant coordination sphere in the cationic $[FeCp(CO)_2]^+$ species (shown by the 3D surface plot to have an accessible site for binding from the nucleophilic solvent, Table 2 of the main paper). Contrastingly, the two Co complexes have large ligands on the metal centre and are far more sterically crowded. These have been calculated to have low affinity for the binding of a solvent molecule.

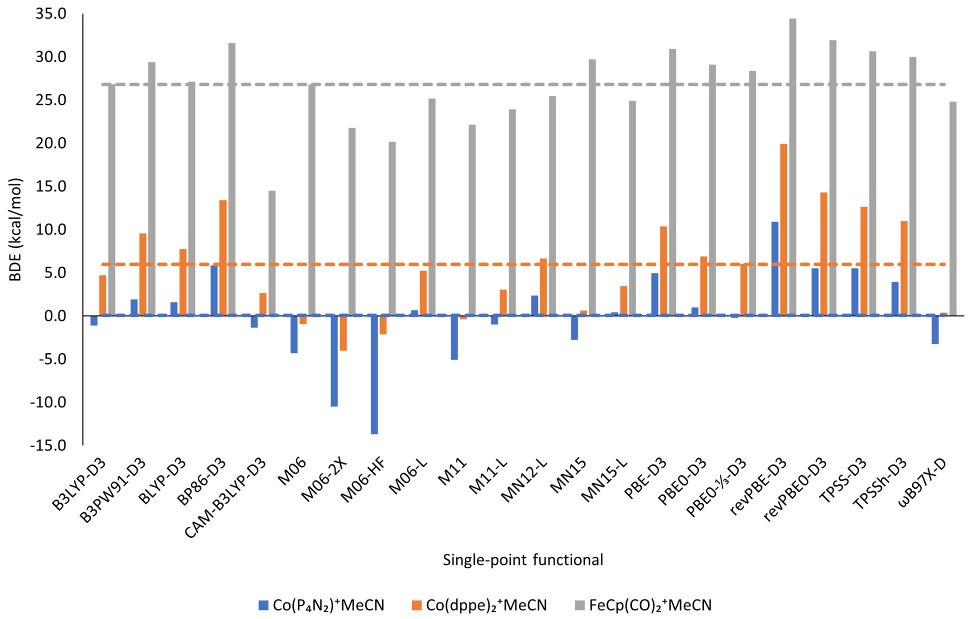


**Figure S2** Graph of the BDE (from Equation S5) for the three complexes examined, across a range of functionals used in the single-point. The dashed line indicates the average BDE across the different single-point functionals. Calculations were performed at the level of functional($PCM_{MeCN}$)/def2-TZVP//RI-BP86-D3($PCM_{MeCN}$)/def2-TZVP.

As such, while the $[FeCp(CO)_2]^+$ cationic species should be coordinated by an explicit solvent molecule (Equation 6), the Co complexes do not require treatment with an explicit solvent molecule (Equation 7 and 8). While there is an element of functional dependence, this methodology has been tested and was found to produce hydricity values closest to experimentally obtained values (Figure S3).

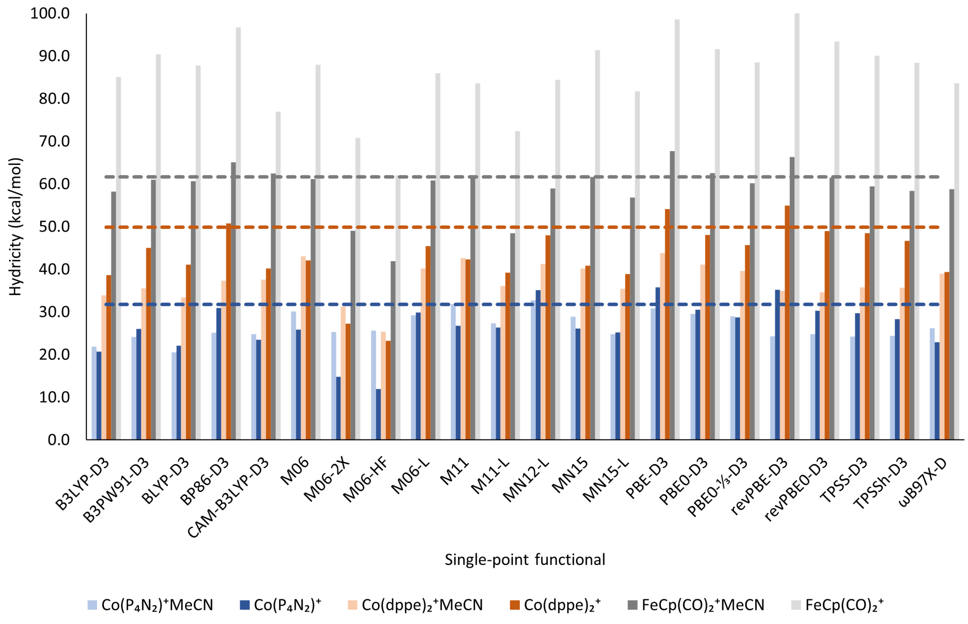


**Figure S3** Graph of the computed hydricity values across the the three complexes examined, using a range of single-point functionals and calculated with both bound, $[ML(MeCN)]^+$, and unbound, $[ML]^+$, forms of the metal cationic complex. The dashed line represents the literature hydricity value for each complex. The darker colour for each complex represents the methodology selected based on the calculated BDE (Figure S2, using Equations 6–8). Calculations were performed at the level of functional($PCM_{MeCN}$)/def2-TZVP//RI-BP86-D3($PCM_{MeCN}$)/def2-TZVP.

# 4 Methodological Considerations

## 4.1 Choice of Single-Point Basis

The single-point basis set was chosen to be a triple-$\zeta$ basis, def2-TZVP. The effect of using a smaller double-$\zeta$ and a larger quadruple-$\zeta$ basis in single-points was considered for the smallest complex, $FeCp(CO)_2H$ and hydricity values were calculated using each basis set. While there was a noticeable difference using the smaller basis, the step up to a quadruple-$\zeta$ basis was found to have a minimal impact upon the computed hydricity values (Figure S4).

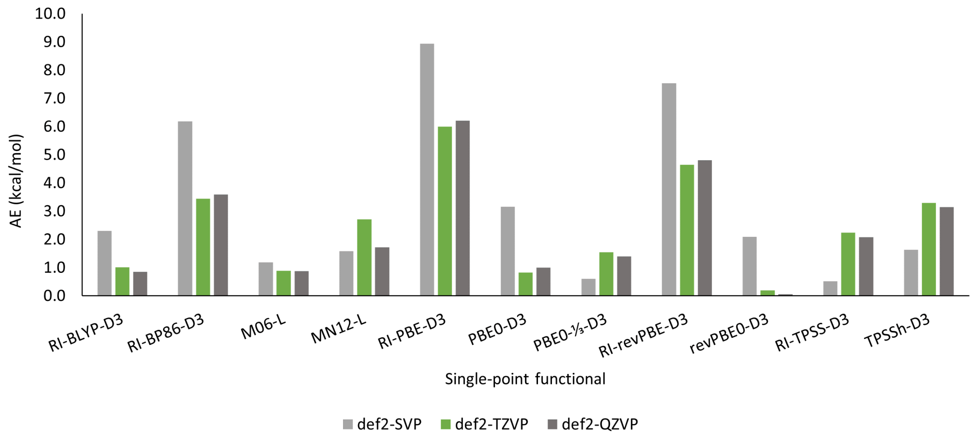


**Figure S4** Absolute error in the calculated hydricity values for $FeCp(CO)_2H$ across a number of functionals using double-, triple- and quadruple-$\zeta$ basis sets. Single-point calculations were performed with implicit solvation on geometries from the level of RI-BP86-D3($PCM_{MeCN}$)/def2-TZVP.

As there was no significant improvement to be found from the use of a larger basis, we recommend that def2-TZVP is of sufficient accuracy without the additional expense associated with using a quadruple-$\zeta$ basis set.

## 4.2 Description of Geometries

### 4.2.1 Choice of Functional

RI-BP86 was initially chosen for use in geometry optimisation based on previous methodology from the group [10]. Additionally, as a pure functional, density fitting can be used to reduce the cost of these calculations, beneficial for systems of this size (Co complexes with *ca.* 100 atoms) where the use of a hybrid functional quickly becomes expensive.

A number of functionals have been used in the literature for the geometry optimisation of 3d metal complexes (Table 1 in the main paper). Four different functionals, BP86, PBE0, M06-L and M06 have been used in the geometry optimisation to compare the effect of using functionals from different levels, pure GGA, hybrid, meta-GGA, and meta-hybrid GGA.

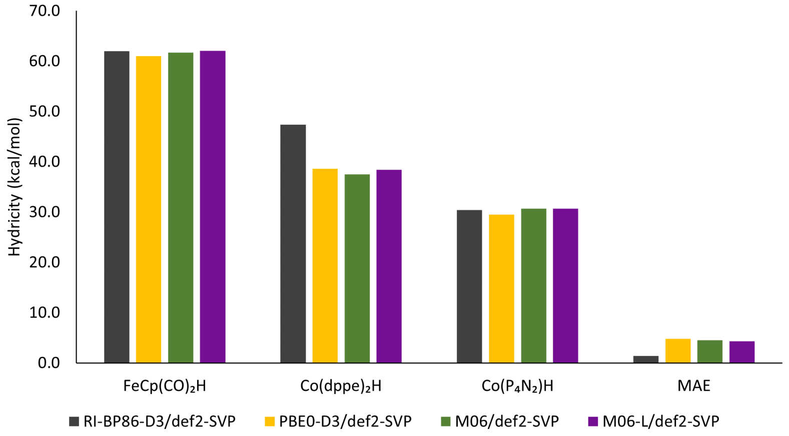


**Figure S5** Calculated hydricity values for the three chosen complexes using different functionals in the geometry optimisation with def2-SVP basis, PCM implicit solvation and D3 dispersion corrections followed by PBE0-D3($PCM_{MeCN}$)/def2-TZVP single-points.

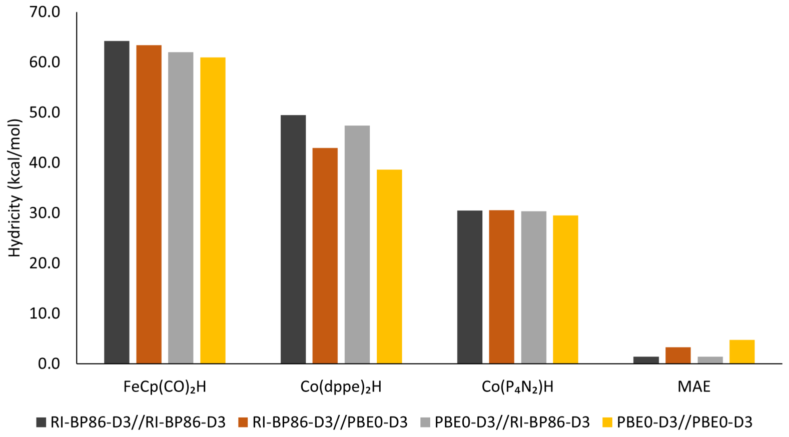


**Figure S6** Calculated hydricity values for the three chosen complexes using different combinations of BP86 and PBE0 in geometry optimisation and single-point calculations. Performed with $PCM_{MeCN}$ implicit solvation in both optimisation and single-point calculations, using def2-SVP and def2-TZVP basis, respectively.

The lowest MAE was obtained with RI-BP86-D3 in the geometry optimisation, which would appear to be best suited for the description of these geometries as the pure functional remains far cheaper than the use of a more expensive hybrid functional in both geometry optimisation and frequency calculations.

### 4.2.2 The Effect of Basis Set in Geometry Optimisation

A selection of basis sets have been compared to determine the most suitable for use in geometry optimisations. In augmentation of the def2-SVP basis, an additional diffuse 's' function was placed on the hydridic hydrogen. Geometry optimisations were performed using each basis set, with a selection of single-points performed to examine the trends across functionals on the calculated value of hydricity. MAEs were calculated for each different basis and functional across the three compounds (Figure S7).

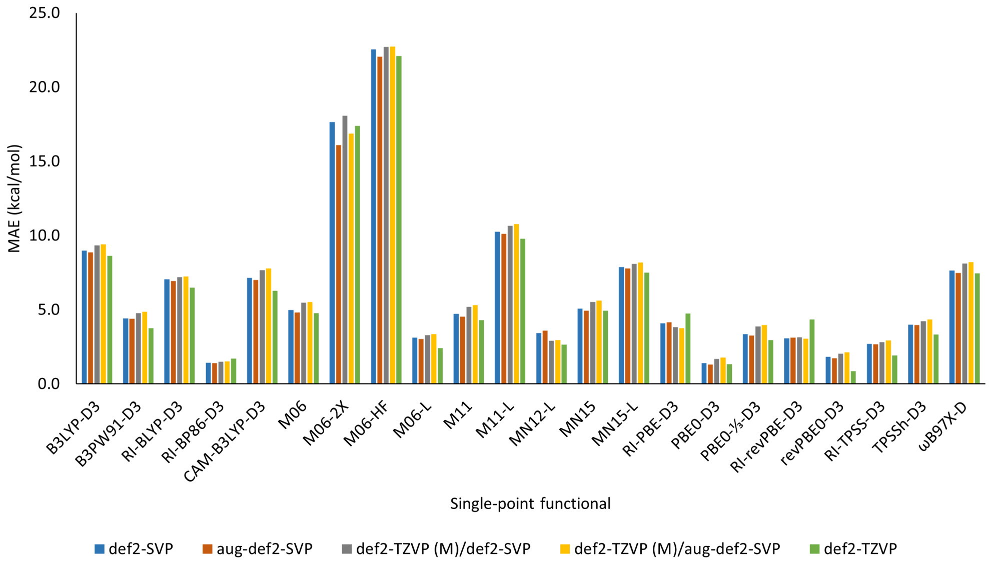


**Figure S7** MAEs for the calculation of hydricity across the three complexes using a range of single-point functionals on geometries obtained using five different combinations of basis sets. Calculations performed at the level of functional($PCM_{MeCN}$)/def2-TZVP//RI-BP86-D3($PCM_{MeCN}$)/geometry basis.

As mentioned in the main paper, it was found that while the largest basis set was found to perform best, there was minimal improvement to be found when a suitable functional was used. As such, we found that with the use of RI-BP86 and PBE0, a smaller double-$\zeta$ basis set was sufficient and offered a good balance between accuracy and expense.

### 4.2.3 Optimisation Conditions

The use of implicit solvation and empirical dispersion has been considered, with inclusion in both geometry optimisation and in single-points (Figure S8).

**Table S2** MAEs obtained from hydricity calculations as shown in Figure S8. Geometry optimisations were performed using the def2-SVP basis set and with the methodology outlined in the table. Further single-point calculations were performed at the level of PBE0-D3($PCM_{MeCN}$)/def2-TZVP.

| Method | RI-BP86-D3(PCM) | RI-BP86(PCM) | RI-BP86-D3 | RI-BP86 |
|---|---|---|---|---|
| MAE (kcal/mol) | 1.41 | 4.32 | 2.45 | 4.67 |

The largest average errors were obtained using methods without dispersion and, in some cases, the inclusion of dispersion was shown to alter the calculated hydricity value by over 5 kcal/mol. As such, these corrections should be included in geometry optimisation where possible. The inclusion of a solvent model in optimisation also has minimal impact upon the overall energetics of the reaction, though the lowest MAE was obtained when both solvation and dispersion were included. In work by Dub and Ikariya [11] and also by our group [12], the inclusion of a solvent model has been shown to influence the asynchronicity of transition states and it is hence strongly recommended for inclusion in geometry optimisations.

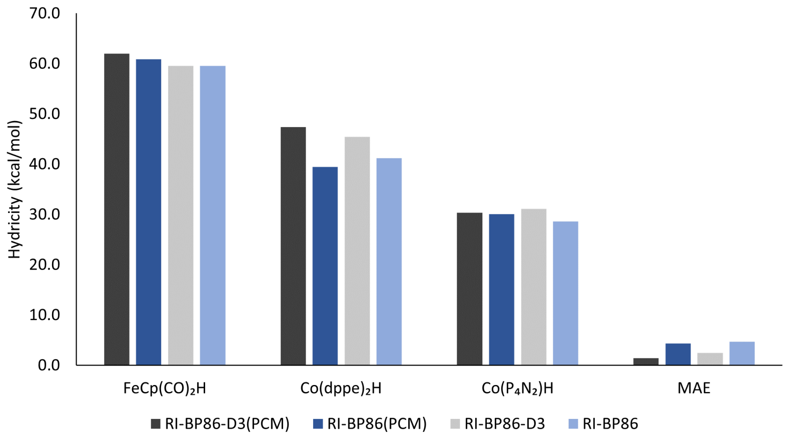


**Figure S8** Calculated hydricity values using a range of different optimisation methodologies with the functional, RI-BP86 and def2-SVP basis. Further single-point calculations were performed at the level of PBE0-D3($PCM_{MeCN}$)/def2-TZVP.

### 4.2.4 Comparison of X-Ray and Optimised Structures

Often benchmarking studies are performed with a focus upon the comparison of bond lengths between optimised structures and X-ray crystal structures. The geometry of the optimised structure Co(dppe)$_2$H at the level of RI-BP86-D3($PCM_{MeCN}$)/def2-SVP, RI-BP86-D3($PCM_{MeCN}$)/def2-TZVP and PBE0-D3($PCM_{MeCN}$)/def2-SVP been compared to the X-Ray crystal structure available from Ciancanelli *et al.* [13] (Co(dppe)$_2$H) and to a related crystal structure from Ariyaratne *et al.* [14] (FeCp(CO)$_2$($CH_2$COOH)) (Table S3). While there is a little deviation in bond lengths between computed and X-Ray structures, this is to be expected as the X-Ray structures are obtained in the solid phase, from thermally averaged distances whereas computations are performed in the gas phase with an implicit solvent model. One of the key considerations was the choice of basis set for geometry optimisations. It was found that using the def2-TZVP basis in the geometry optimisation produced more accurate energetics in the calculation of hydricity of the three complexes; however, def2-SVP performed well for a lower cost. As shown in Table S3, the use of a larger basis set had very little effect upon the central bond distances of the complex and the double-$\zeta$ basis set may be judged to perform suitably well for use in geometry optimisations.

**Table S3** Selected bond lengths (Å) of Co(dppe)$_2$H and FeCp(CO)$_2$H in computed structures and X-Ray from Ciancanelli *et al.* [13] and Ariyaratne *et al.* [14].

| Method | Co−H[a] | Co−P[b] | Fe−C(Cp)[c] | Fe−C(CO)[d] |
|---|---|---|---|---|
| X-Ray [13, 14] | 1.46(3) | 2.148 | 2.163 | 1.730 |
| RI-BP86-D3(PCM)/def2-SVP | 1.53 | 2.129 | 2.109 | 1.736 |
| RI-BP86-D3(PCM)/def2-TZVP | 1.53 | 2.126 | 2.115 | 1.740 |
| PBE0-D3(PCM)/def2-SVP | 1.51 | 2.136 | 2.088 | 1.737 |

[a] Bond distances involving hydrogen are commonly found to differ between X-Ray and optimised (equilibrium) structures as the positions of light elements, such as hydrogen, are notoriously difficult to refine using X-Ray diffraction.
[b] Mean of the Co−P bond distances to each of the 4 phosphorus atoms of the two dppe ligands.
[c] Mean of the Fe−C bond distances to each of the 5 carbon atoms of the Cp ring.
[d] Mean of the Fe−C bond distances to the two carbonyl ligands.

## 4.3 Solvation Model

There are a variety of implicit solvation methods available and the effect of using IEF-PCM, C-PCM and SMD has been compared (Table S4).

**Table S4** MAE for the hydricity values using a variety of implicit solvation methods in single-point calculations. Calculations were performed at the level of PBE0-D3(implicit solvation$_{\mathrm{MeCN}}$)/def2-TZVP//RI-BP86-D3(PCM$_{\mathrm{MeCN}}$)/def2-SVP.

| Implicit Solvation Method | MAE (kcal/mol) |
|---|---|
| IEF-PCM | 1.4 |
| C-PCM | 1.3 |
| SMD | 0.9 |

With only a difference of 0.5 kcal/mol between the different forms of implicit solvation, there is no real notable difference in the solvation method used, and a far greater emphasis is placed on simply the inclusion of implicit solvation.

## 4.4 Use of Different Gaussian Versions

To ensure consistency between the use of different versions of Gaussian, thermodynamic hydricity was evaluated at the chosen level of PBE0-D3(PCM$_{\mathrm{MeCN}}$)/def2-TZVP//RI-BP86-D3(PCM$_{\mathrm{MeCN}}$)/def2-SVP using Gaussian 09, Gaussian 16 using the defaults of Gaussian 09 and using Gaussian 16. These have been evaluated to 5 significant figures, which while of limited physical value, give good indication of the reproducibility of results across different versions of Gaussian (Table S5).

**Table S5** Thermodynamic hydricity ($\Delta G_{\mathrm{H}^-}$) in kcal/mol for each of the three complexes using different varieties of Gaussian at the level of PBE0-D3(PCM$_{\mathrm{MeCN}}$)/def2-TZVP//RI-BP86-D3(PCM$_{\mathrm{MeCN}}$)/def2-SVP.

| Complex | G09 | G16 with G09 defaults | G16 |
|---|---|---|---|
| $Co(dppe)_2H$ | 47.405 | 47.405 | 47.400 |
| $Co(P_4N_2)H$ | 30.368 | 30.368 | 30.367 |
| $FeCp(CO)_2H$ | 61.996 | 61.996 | 61.994 |

It was found that the energies were accurately reproduced across different versions of the programme. Where Gaussian 16 was required for use in this work, the keyword G09Defaults was always used.

## 4.5 Hydride Binding Energy

The thermodynamic hydricity has been evaluated at 298.15K to allow for comparison to the experimentally derived data from Wiedner *et al.* [15]. To facilitate reproducibility, in Table S6 these hydricity values are broken down for one illustrative level into raw potential energies (at 0 K), enthalpies and Gibbs free energies (for Equations 6–8 in the main paper).

**Table S6** Thermodynamic properties for each of the three complexes using the level of PBE0-D3(PCM$_{\mathrm{MeCN}}$)/def2-TZVP//RI-BP86-D3(PCM$_{\mathrm{MeCN}}$)/def2-SVP. All values are provided in units of kcal/mol except $\Delta S$ which is given in units of cal/(K mol).

| Complex | $\Delta E_{opt}$ | $\Delta E_{sp}$ | $\Delta S$ | $\Delta H$ | $\Delta G$ | $\Delta G_{\mathrm{H}^-}$ |
|---|---|---|---|---|---|---|
| $Co(dppe)_2H$ | -12.3 | -21.5 | 43.7 | -22.8 | -35.9 | 47.4 |
| $Co(P_4N_2)H$ | -32.8 | -36.3 | 52.7 | -37.2 | -52.9 | 30.4 |
| $FeCp(CO)_2H$ | -6.8 | -11.7 | 16.8 | -12.6 | -17.6 | 62.0 |

## References


1 Ryu, H.; Park, J.; Kim, H.K.; Park, J.Y.; Kim, S.T.; Baik, M.H. Pitfalls in Computational Modeling of Chemical Reactions and How to Avoid Them. *Organometallics* **2018**, *37*, 3228–3239. doi:10.1021/acs.organomet.8b00456.

2 Martin, R.L.; Hay, P.J.; Pratt, L.R. Hydrolysis of ferric ion in water and conformational equilibrium. *J. Phys. Chem. A* **1998**, *102*, 3565–3573. doi:10.1021/jp980229p.

3 Bühl, M.; Sieffert, N.; Wipff, G. Density functional study of aqueous uranyl(VI) fluoride complexes. *Chem. Phys. Lett.* **2009**, *467*, 287–293. doi:10.1016/j.cplett.2008.11.015.

4 Bühl, M.; Sieffert, N.; Chaumont, A.; Wipff, G. Water versus acetonitrile coordination to uranyl. Effect of chloride ligands. *Inorg. Chem.* **2012**, *51*, 1943–1952. doi:10.1021/ic202270u.

5 Sieffert, N.; Bühl, M. Noncovalent interactions in a transition-metal triphenylphosphine complex: A density functional case study. *Inorg. Chem.* **2009**, *48*, 4622–4624. doi:10.1021/ic900347e.

6 Sieffert, N.; Bühl, M. Hydrogen generation from alcohols catalyzed by ruthenium-triphenylphosphine complexes: Multiple reaction pathways. *J. Am. Chem. Soc.* **2010**, *132*, 8056–8070. doi:10.1021/ja101044c.

7 Sieffert, N.; Réocreux, R.; Lorusso, P.; Cole-Hamilton, D.J.; Bühl, M. On the importance of decarbonylation as a side-reaction in the ruthenium-catalysed dehydrogenation of alcohols: A combined experimental and density functional study. *Chem. Eur. J.* **2014**, *20*, 4141–4155. doi:10.1002/chem.201303722.

8 Gallarati, S.; Dingwall, P.; Fuentes, J.A.; Bühl, M.; Clarke, M.L. Understanding Catalyst Structure-Selectivity Relationships in Pd-Catalyzed Enantioselective Methoxycarbonylation of Styrene. *Organometallics* **2020**, *39*, 4544–4556. doi:10.1021/acs.organomet.0c00613.

9 Himmel, D.; Goll, S.K.; Leito, I.; Krossing, I. A unified pH scale for all phases. *Angew. Chemie - Int. Ed.* **2010**, *49*, 6885–6888. doi:10.1002/anie.201000252.

10 Bühl, M.; Kabrede, H. Geometries of transition-metal complexes from density-functional theory. *J. Chem. Theory Comput.* **2006**, *2*, 1282–1290. doi:10.1021/ct800172j.

11 Dub, P.A.; Ikariya, T. Quantum chemical calculations with the inclusion of nonspecific and specific solvation: Asymmetric transfer hydrogenation with bifunctional ruthenium catalysts. *J. Am. Chem. Soc.* **2013**, *135*, 2604–2619. doi:10.1021/ja3097674.

12 Goodfellow, A.S.; Owen, A.E.; Bühl, M. Unpublished Calculations **2020**.

13 Ciancanelli, R.; Noll, B.C.; DuBois, D.L.; Rakowski DuBois, M. Comprehensive thermodynamic characterization of the metal-hydrogen bond in a series of cobalt-hydride complexes. *J. Am. Chem. Soc.* **2002**, *124*, 2984–2992. doi:10.1021/ja0122804.

14 Ariyaratne, J.K.P.; Bierrum, A.M.; Green, M.L.H.; Ishaq, M.; Prout, C.K.; Swanwick, M.G. Evidence for near-neighbour interactions in some substituted methyl derivatives of transition metals including the molecular crystal structure determinations of ($\pi$-$C_5H_5$)$Fe(CO)_2CH_2CO_2H$ and ($\pi$-$C_5H_5$)$Mo(CO)_3CH_2CO_2H$. *J. Chem. Soc. A* **1969**, pp. 1309–1321. doi:10.1039/J19690001309.

15 Wiedner, E.S.; Chambers, M.B.; Pitman, C.L.; Bullock, R.M.; Miller, A.J.; Appel, A.M. Thermodynamic Hydricity of Transition Metal Hydrides. *Chem. Rev.* **2016**, *116*, 8655–8692. doi:10.1021/acs.chemrev.6b00168.

# 5 Cartesian Coordinates of Optimised Structures

Raw data and cartesian coordinates obtained from geometry optimisation and frequency calculations at the level of RI-BP86-D3($PCM_{MeCN}$)/def2-SVP.

```
Codppe2H
Frequencies, energies and thermodynamic parameters:
 Lowest Vibrational Mode (1/cm) =                            8.8629
 E(RB-P86) (a.u.) =                                         -4758.00249090
 Thermal correction to Enthalpy (a.u.) =                     0.887329
 Thermal correction to Gibbs Free Energy (a.u.) =            0.743101
 Total Entropy (cal/Kmol) =                                  303.552
Optimised cartesian coordinates (Angstrom):
P      1.533305   0.883124   1.012500
P     -0.763931   1.975020  -0.306669
P     -1.570628  -0.892874   1.004569
P      0.773930  -1.970753  -0.308995
C      0.843648   2.383321   1.954113
C     -0.518656   2.786843   1.364058
C      2.938838   1.692248   0.091414
C      2.862051   1.717386  -1.312202
C      3.841710   2.373528  -2.074425
C      4.914313   3.014423  -1.429723
C      4.999227   2.997385  -0.023382
C      4.014510   2.340894   0.734788
C      2.461098  -0.064355   2.292897
C      3.684743  -0.696117   1.977135
C      4.287959  -1.579292   2.888576
C      3.684776  -1.842733   4.131096
C      2.468808  -1.212202   4.458877
C      1.863214  -0.337170   3.544124
C      0.057685   3.131293  -1.498671
C      0.053810   2.786771  -2.868527
C      0.728643   3.580760  -3.806930
C      1.435027   4.724994  -3.386951
C      1.444838   5.073637  -2.026878
C      0.754756   4.285568  -1.088273
C     -2.540407   2.303262  -0.691562
C     -3.268926   3.358323  -0.102592
C     -4.641529   3.501562  -0.367905
C     -5.294570   2.601194  -1.230296
C     -4.565449   1.564569  -1.841434
C     -3.195422   1.418671  -1.570911
C     -0.896320  -2.429656   1.901420
C      0.493123  -2.790830   1.352627
C     -3.012720  -1.673778   0.111456
C     -2.916772  -1.800252  -1.286035
C     -3.919591  -2.453741  -2.020530
C     -5.039707  -2.983602  -1.355829
C     -5.146363  -2.861471   0.043754
C     -4.135214  -2.214512   0.775071
C     -2.457880   0.057723   2.312405
C     -1.891195   0.238373   3.594754
C     -2.453448   1.137969   4.514466
C     -3.596953   1.881845   4.166092
C     -4.178430   1.700345   2.898391
C     -3.618821   0.795507   1.981406
C     -0.014165  -3.123053  -1.528481
```

| | | | |
|---|---|---|---|
| C | -0.033771 | -2.741988 | -2.888659 |
| C | -0.681623 | -3.537224 | -3.844919 |
| C | -1.337581 | -4.720829 | -3.453140 |
| C | -1.324215 | -5.106173 | -2.103038 |
| C | -0.660101 | -4.316274 | -1.146823 |
| C | 2.561798 | -2.312369 | -0.644635 |
| C | 3.222058 | -1.499865 | -1.588516 |
| C | 4.592779 | -1.666032 | -1.842633 |
| C | 5.321034 | -2.651630 | -1.150911 |
| C | 4.665305 | -3.479390 | -0.221811 |
| C | 3.292256 | -3.314503 | 0.028401 |
| H | 0.743998 | 2.111200 | 3.023574 |
| H | 1.583756 | 3.206186 | 1.892660 |
| H | -0.647415 | 3.886981 | 1.324653 |
| H | -1.346561 | 2.380219 | 1.977710 |
| H | 1.993898 | 1.220958 | -1.776273 |
| H | 3.759808 | 2.393762 | -3.172310 |
| H | 5.686025 | 3.532988 | -2.020812 |
| H | 5.838866 | 3.499431 | 0.483816 |
| H | 4.088875 | 2.327940 | 1.834773 |
| H | 4.160477 | -0.506988 | 1.003304 |
| H | 5.234119 | -2.072834 | 2.616713 |
| H | 4.158208 | -2.538029 | 4.841840 |
| H | 1.985473 | -1.408689 | 5.429000 |
| H | 0.899265 | 0.129096 | 3.795974 |
| H | -0.457082 | 1.864003 | -3.188786 |
| H | 0.717299 | 3.299022 | -4.871932 |
| H | 1.980928 | 5.338769 | -4.120486 |
| H | 2.000995 | 5.961605 | -1.687164 |
| H | 0.784574 | 4.574025 | -0.027347 |
| H | -2.776707 | 4.062880 | 0.584118 |
| H | -5.208046 | 4.315660 | 0.111252 |
| H | -6.373236 | 2.708614 | -1.425421 |
| H | -5.068080 | 0.854043 | -2.516508 |
| H | -2.620627 | 0.591396 | -2.009041 |
| H | -0.848889 | -2.222325 | 2.988968 |
| H | -1.622655 | -3.254187 | 1.757488 |
| H | 0.652846 | -3.887288 | 1.317927 |
| H | 1.288908 | -2.363418 | 1.994818 |
| H | -2.024610 | -1.377023 | -1.774395 |
| H | -3.821059 | -2.552967 | -3.112921 |
| H | -5.832087 | -3.494947 | -1.925479 |
| H | -6.022997 | -3.275570 | 0.567585 |
| H | -4.224327 | -2.124794 | 1.870156 |
| H | -0.993186 | -0.329336 | 3.881240 |
| H | -1.993093 | 1.260868 | 5.507675 |
| H | -4.033879 | 2.596205 | 4.881259 |
| H | -5.073114 | 2.275010 | 2.611618 |
| H | -4.080199 | 0.672596 | 0.990385 |
| H | 0.432346 | -1.788835 | -3.186513 |
| H | -0.689240 | -3.225699 | -4.901593 |
| H | -1.862395 | -5.336548 | -4.200321 |
| H | -1.840739 | -6.025477 | -1.784673 |
| H | -0.670738 | -4.635028 | -0.094023 |
| H | 2.653001 | -0.712259 | -2.100770 |
| H | 5.096697 | -1.012629 | -2.572213 |
| H | 6.399745 | -2.774955 | -1.335926 |

```
H      5.229232  -4.253460   0.322333
H      2.801854  -3.962873   0.768890
Co     -0.015248  -0.002902  -0.156986
H     -0.107759  -0.002809  -1.681462
```

Codppe2+MeCN

```
Frequencies, energies and thermodynamic parameters:
 Lowest Vibrational Mode (1/cm) =                          2.7211
 E(RB-P86) (a.u.) =                                       -4889.99302629
 Thermal correction to Enthalpy (a.u.) =                   0.933193
 Thermal correction to Gibbs Free Energy (a.u.) =          0.779797
 Total Entropy (cal/Kmol) =                                322.849
Optimised cartesian coordinates (Angstrom):
P      1.591034   0.963784   1.040402
P     -0.910376   1.984934   0.007376
P     -1.593250  -0.964398   1.050420
P      0.908251  -1.993048   0.013669
C      0.870663   2.342950   2.127530
C     -0.527351   2.764357   1.647634
C      2.851367   1.895463   0.047878
C      2.751134   1.872213  -1.355701
C      3.650944   2.599281  -2.150377
C      4.654510   3.372462  -1.542868
C      4.753182   3.415104  -0.138793
C      3.857682   2.678797   0.654252
C      2.642671   0.060718   2.258222
C      3.992430  -0.258302   2.002456
C      4.728869  -1.023856   2.921124
C      4.130870  -1.481205   4.107570
C      2.784822  -1.167916   4.373079
C      2.048241  -0.406922   3.452261
C     -0.247568   3.187968  -1.223957
C     -0.638440   3.078501  -2.577261
C     -0.069933   3.914519  -3.551332
C      0.889659   4.876437  -3.185207
C      1.261865   5.009479  -1.837469
C      0.699349   4.170086  -0.862446
C     -2.717560   2.200176  -0.231543
C     -3.453955   3.175198   0.472464
C     -4.837414   3.294217   0.263477
C     -5.492012   2.451830  -0.653358
C     -4.756275   1.493025  -1.372471
C     -3.373915   1.368866  -1.161867
C     -0.878251  -2.351426   2.130877
C      0.517844  -2.776942   1.650195
C     -2.867531  -1.884727   0.064693
C     -2.767059  -1.872522  -1.338865
C     -3.678082  -2.590700  -2.128917
C     -4.693190  -3.344443  -1.516306
C     -4.791768  -3.376996  -0.111834
C     -3.885061  -2.649369   0.676376
C     -2.631231  -0.055539   2.275952
C     -2.036578   0.390490   3.478565
C     -2.765638   1.157193   4.400658
C     -4.103904   1.497640   4.129134
C     -4.701965   1.061884   2.934760
C     -3.973050   0.291472   2.014099
```

| | | | |
|---|---|---|---|
| C | 0.252970 | -3.193258 | -1.223841 |
| C | 0.660882 | -3.088579 | -2.572503 |
| C | 0.090125 | -3.913030 | -3.554934 |
| C | -0.885650 | -4.862880 | -3.200428 |
| C | -1.273024 | -4.994103 | -1.856618 |
| C | -0.710981 | -4.163179 | -0.874098 |
| C | 2.717628 | -2.205122 | -0.213165 |
| C | 3.379679 | -1.375585 | -1.141188 |
| C | 4.763305 | -1.499936 | -1.343249 |
| C | 5.495335 | -2.455890 | -0.616738 |
| C | 4.835696 | -3.294971 | 0.299381 |
| C | 3.450746 | -3.176922 | 0.498740 |
| H | 0.831194 | 1.957325 | 3.164255 |
| H | 1.585347 | 3.189472 | 2.129041 |
| H | -0.640161 | 3.864187 | 1.584863 |
| H | -1.309156 | 2.386937 | 2.335218 |
| H | 1.932360 | 1.302791 | -1.815671 |
| H | 3.553653 | 2.575927 | -3.246516 |
| H | 5.358679 | 3.950599 | -2.161475 |
| H | 5.535566 | 4.023674 | 0.341309 |
| H | 3.950683 | 2.711263 | 1.751714 |
| H | 4.470975 | 0.078488 | 1.072428 |
| H | 5.777957 | -1.271574 | 2.698298 |
| H | 4.710082 | -2.083237 | 4.824664 |
| H | 2.302797 | -1.521050 | 5.297736 |
| H | 0.989762 | -0.185585 | 3.659979 |
| H | -1.408246 | 2.349245 | -2.870443 |
| H | -0.385459 | 3.819634 | -4.602213 |
| H | 1.339420 | 5.527587 | -3.950640 |
| H | 2.006918 | 5.762523 | -1.538752 |
| H | 1.025168 | 4.283034 | 0.180796 |
| H | -2.961115 | 3.835559 | 1.200812 |
| H | -5.410141 | 4.044561 | 0.830124 |
| H | -6.578561 | 2.542874 | -0.806308 |
| H | -5.259691 | 0.827550 | -2.090646 |
| H | -2.802949 | 0.603375 | -1.702921 |
| H | -0.837923 | -1.972310 | 3.169936 |
| H | -1.597108 | -3.194429 | 2.126913 |
| H | 0.625258 | -3.876959 | 1.581652 |
| H | 1.300916 | -2.407045 | 2.340431 |
| H | -1.940056 | -1.317576 | -1.802137 |
| H | -3.580937 | -2.575541 | -3.225234 |
| H | -5.406659 | -3.915219 | -2.131084 |
| H | -5.583131 | -3.970663 | 0.372205 |
| H | -3.977859 | -2.673472 | 1.774060 |
| H | -0.984290 | 0.148319 | 3.694988 |
| H | -2.283662 | 1.493106 | 5.331718 |
| H | -4.676938 | 2.103603 | 4.847878 |
| H | -5.744867 | 1.330394 | 2.707057 |
| H | -4.450948 | -0.028061 | 1.077540 |
| H | 1.446519 | -2.372276 | -2.854620 |
| H | 0.415225 | -3.816325 | -4.602535 |
| H | -1.336830 | -5.506070 | -3.971772 |
| H | -2.030659 | -5.738223 | -1.567299 |
| H | -1.049999 | -4.273079 | 0.165356 |
| H | 2.812963 | -0.610422 | -1.687213 |
| H | 5.270606 | -0.836274 | -2.060361 |

```
H      6.582855  -2.546695  -0.762712
H      5.405397  -4.042527   0.872727
H      2.954212  -3.835815   1.225898
Co     -0.001953  -0.004227  -0.079665
N      0.000500  -0.011256  -1.936740
C      0.009783  -0.030018  -3.114960
C      0.026322  -0.061830  -4.565889
H     -0.053327   0.968265  -4.969722
H      0.974085  -0.507443  -4.932444
H     -0.820694  -0.663500  -4.955889
```

```
Codppe2+
Frequencies, energies and thermodynamic parameters:
 Lowest Vibrational Mode (1/cm) =                          16.7535
 E(RB-P86) (a.u.) =                                       -4757.28056961
 Thermal correction to Enthalpy (a.u.) =                   0.882165
 Thermal correction to Gibbs Free Energy (a.u.) =          0.741488
 Total Entropy (cal/Kmol) =                                296.081
Optimised cartesian coordinates (Angstrom):
P      1.646419   1.244459   0.793899
P     -1.105346   1.850575   0.132144
P     -1.646230  -1.247717   0.788565
P      1.105678  -1.851754   0.124968
C      1.006346   2.790434   1.683051
C     -0.514876   2.923283   1.524783
C      2.773353   1.970967  -0.470913
C      2.581493   1.603082  -1.816040
C      3.359587   2.173502  -2.834669
C      4.340299   3.127168  -2.512266
C      4.531835   3.512028  -1.171956
C      3.749998   2.941006  -0.154121
C      2.754299   0.479722   2.051086
C      4.155157   0.409435   1.927684
C      4.915331  -0.246165   2.910992
C      4.286837  -0.837317   4.019756
C      2.886579  -0.774178   4.145782
C      2.125085  -0.120782   3.165073
C     -0.560170   2.671178  -1.424391
C     -0.656170   1.935044  -2.628634
C     -0.159653   2.463357  -3.828951
C      0.460798   3.725979  -3.837338
C      0.569843   4.458650  -2.643273
C      0.058235   3.938361  -1.442275
C     -2.931776   2.069511   0.131875
C     -3.598913   2.825629   1.117487
C     -4.998396   2.945706   1.088730
C     -5.745257   2.314979   0.080324
C     -5.084974   1.562713  -0.908005
C     -3.687877   1.444027  -0.883569
C     -1.006941  -2.798156   1.670585
C      0.514168  -2.931903   1.511495
C     -2.773633  -1.967112  -0.479788
C     -2.580855  -1.592870  -1.823016
C     -3.359121  -2.157412  -2.844758
C     -4.341082  -3.111494  -2.527439
C     -4.533558  -3.502729  -1.189105
C     -3.751460  -2.937635  -0.168159
```

| | | | |
|---|---|---|---|
| C | -2.753803 | -0.489427 | 2.049954 |
| C | -2.124449 | 0.103472 | 3.167972 |
| C | -2.885802 | 0.750827 | 4.152776 |
| C | -4.286055 | 0.815233 | 4.027094 |
| C | -4.914666 | 0.231486 | 2.914499 |
| C | -4.154608 | -0.417816 | 1.926909 |
| C | 0.560384 | -2.664235 | -1.435828 |
| C | 0.657855 | -1.922167 | -2.636316 |
| C | 0.160753 | -2.443336 | -3.839500 |
| C | -0.461736 | -3.704886 | -3.854596 |
| C | -0.572152 | -4.443526 | -2.664365 |
| C | -0.059988 | -3.930372 | -1.460533 |
| C | 2.932020 | -2.071425 | 0.123669 |
| C | 3.688533 | -1.439934 | -0.887750 |
| C | 5.085513 | -1.559633 | -0.913190 |
| C | 5.745333 | -2.318674 | 0.070250 |
| C | 4.998126 | -2.955000 | 1.074867 |
| C | 3.598721 | -2.834030 | 1.104567 |
| H | 1.294250 | 2.714793 | 2.749327 |
| H | 1.537866 | 3.663493 | 1.257475 |
| H | -0.839836 | 3.975659 | 1.395759 |
| H | -1.036614 | 2.521509 | 2.416351 |
| H | 1.781696 | 0.888907 | -2.059435 |
| H | 3.188230 | 1.881999 | -3.881833 |
| H | 4.951235 | 3.581441 | -3.307775 |
| H | 5.294008 | 4.264959 | -0.917429 |
| H | 3.904776 | 3.257026 | 0.889754 |
| H | 4.656474 | 0.842626 | 1.051002 |
| H | 6.008461 | -0.307252 | 2.798074 |
| H | 4.886591 | -1.357168 | 4.782806 |
| H | 2.384371 | -1.239450 | 5.008154 |
| H | 1.026656 | -0.084930 | 3.248483 |
| H | -1.099763 | 0.927351 | -2.623957 |
| H | -0.240543 | 1.877154 | -4.757213 |
| H | 0.868600 | 4.134581 | -4.774618 |
| H | 1.063363 | 5.442704 | -2.641784 |
| H | 0.154726 | 4.526649 | -0.517836 |
| H | -3.041374 | 3.314185 | 1.927868 |
| H | -5.507061 | 3.529756 | 1.871058 |
| H | -6.842462 | 2.405190 | 0.066338 |
| H | -5.659298 | 1.060492 | -1.701783 |
| H | -3.187801 | 0.846687 | -1.657487 |
| H | -1.294542 | -2.726885 | 2.737236 |
| H | -1.539440 | -3.668891 | 1.241478 |
| H | 0.837771 | -3.983859 | 1.375738 |
| H | 1.036586 | -2.536729 | 2.405673 |
| H | -1.780242 | -0.878282 | -2.062448 |
| H | -3.186994 | -1.860928 | -3.890395 |
| H | -4.952259 | -3.561114 | -3.325402 |
| H | -5.296692 | -4.256029 | -0.938577 |
| H | -3.907047 | -3.258685 | 0.874068 |
| H | -1.026084 | 0.066264 | 3.251724 |
| H | -2.383517 | 1.210214 | 5.018252 |
| H | -4.885675 | 1.330190 | 4.793563 |
| H | -6.007773 | 0.293589 | 2.801910 |
| H | -4.656047 | -0.845095 | 1.047390 |
| H | 1.103077 | -0.915268 | -2.626119 |

| | | | |
|---|---|---|---|
| H | 0.242805 | -1.852358 | -4.764627 |
| H | -0.870072 | -4.107944 | -4.794041 |
| H | -1.067217 | -5.426801 | -2.668115 |
| H | -0.157910 | -4.523489 | -0.539338 |
| H | 3.188950 | -0.836837 | -1.657478 |
| H | 5.660129 | -1.052799 | -1.703818 |
| H | 6.842457 | -2.409748 | 0.055474 |
| H | 5.506440 | -3.544292 | 1.853485 |
| H | 3.040830 | -3.327095 | 1.911982 |
| Co | 0.000260 | -0.000604 | 0.193496 |

CoP4N2H

Frequencies, energies and thermodynamic parameters:

| | |
|---|---|
| Lowest Vibrational Mode (1/cm) = | 19.0970 |
| E(RB-P86) (a.u.) = | -4561.52443865 |
| Thermal correction to Enthalpy (a.u.) = | 0.826463 |
| Thermal correction to Gibbs Free Energy (a.u.) = | 0.699617 |
| Total Entropy (cal/Kmol) = | 266.970 |

Optimised cartesian coordinates (Angstrom):

| | | | |
|---|---|---|---|
| P | 1.158047 | -1.274166 | -1.248920 |
| P | -1.756847 | -1.126535 | -1.000791 |
| P | -1.755619 | 0.153332 | 1.457087 |
| P | 1.157436 | 0.505171 | 1.537664 |
| N | -3.714661 | -1.732245 | 0.884560 |
| N | -2.800946 | 1.443144 | -0.799623 |
| C | 1.415852 | -4.099827 | -1.334865 |
| H | 0.445476 | -4.096215 | -1.853935 |
| C | 2.011984 | -5.332381 | -1.021623 |
| H | 1.509202 | -6.269441 | -1.309430 |
| C | 3.245528 | -5.370366 | -0.344367 |
| H | 3.713791 | -6.336332 | -0.097938 |
| C | 3.871415 | -4.164489 | 0.016726 |
| H | 4.833420 | -4.176014 | 0.553043 |
| C | 3.274029 | -2.932064 | -0.295836 |
| H | 3.773723 | -2.000189 | -0.000440 |
| C | 2.042600 | -2.881506 | -0.984311 |
| C | 3.741971 | -0.545531 | -2.377480 |
| H | 3.966053 | -1.614676 | -2.511980 |
| C | 4.703443 | 0.419106 | -2.729935 |
| H | 5.675607 | 0.097165 | -3.136208 |
| C | 4.432145 | 1.790178 | -2.559912 |
| H | 5.194420 | 2.539262 | -2.826977 |
| C | 3.184940 | 2.198615 | -2.051399 |
| H | 2.955336 | 3.267704 | -1.918421 |
| C | 2.232866 | 1.234403 | -1.689303 |
| H | 1.261117 | 1.518644 | -1.257235 |
| C | 2.506423 | -0.140827 | -1.831439 |
| C | 0.220551 | -1.586586 | -2.863080 |
| H | 0.249943 | -0.612977 | -3.394358 |
| H | 0.762218 | -2.322327 | -3.493130 |
| C | -1.222919 | -1.998040 | -2.567966 |
| H | -1.311713 | -3.088383 | -2.378517 |
| H | -1.917598 | -1.753709 | -3.398537 |
| C | -2.922987 | -2.374410 | -0.156889 |
| H | -3.569656 | -2.889493 | -0.888911 |
| H | -2.250056 | -3.131202 | 0.302473 |
| C | -5.801269 | -0.691376 | 1.692910 |

| | | | |
|---|---|---|---|
| H | -5.312632 | -0.332565 | 2.608009 |
| C | -7.161137 | -0.397530 | 1.516323 |
| H | -7.680913 | 0.176363 | 2.300392 |
| C | -7.857312 | -0.813431 | 0.367877 |
| H | -8.922505 | -0.573216 | 0.232627 |
| C | -7.148782 | -1.538491 | -0.605993 |
| H | -7.658545 | -1.878556 | -1.521840 |
| C | -5.788962 | -1.841402 | -0.445611 |
| H | -5.289815 | -2.403516 | -1.245330 |
| C | -5.069687 | -1.431307 | 0.716606 |
| C | -2.939931 | -1.223192 | 2.007286 |
| H | -2.274341 | -2.023390 | 2.397721 |
| H | -3.597916 | -0.887291 | 2.827164 |
| C | -2.985649 | 1.384820 | 0.640662 |
| H | -4.031709 | 1.099353 | 0.885086 |
| H | -2.786653 | 2.373357 | 1.092263 |
| C | -2.128408 | 3.791897 | -0.964032 |
| H | -2.801428 | 4.047269 | -0.132014 |
| C | -1.410643 | 4.809928 | -1.606221 |
| H | -1.532295 | 5.848533 | -1.258531 |
| C | -0.555686 | 4.524272 | -2.688046 |
| H | 0.014053 | 5.326529 | -3.180802 |
| C | -0.444220 | 3.191181 | -3.122619 |
| H | 0.233132 | 2.934133 | -3.952322 |
| C | -1.165838 | 2.164987 | -2.495696 |
| H | -1.018329 | 1.129314 | -2.825375 |
| C | -2.022283 | 2.440362 | -1.396539 |
| C | -3.038625 | 0.193530 | -1.491409 |
| H | -2.992688 | 0.348807 | -2.585618 |
| H | -4.063939 | -0.157860 | -1.249846 |
| C | -1.127561 | 0.975487 | 3.021074 |
| H | -1.183130 | 2.071139 | 2.845649 |
| H | -1.780313 | 0.737903 | 3.886665 |
| C | 0.324988 | 0.534140 | 3.232182 |
| H | 0.365545 | -0.495698 | 3.637635 |
| H | 0.868988 | 1.210828 | 3.922830 |
| C | 2.637469 | 2.862142 | 2.298591 |
| H | 3.231325 | 2.202588 | 2.950722 |
| C | 2.902233 | 4.241224 | 2.287210 |
| H | 3.701909 | 4.651191 | 2.924861 |
| C | 2.145651 | 5.096710 | 1.463954 |
| H | 2.355655 | 6.178035 | 1.449097 |
| C | 1.117602 | 4.565833 | 0.666878 |
| H | 0.512859 | 5.223232 | 0.025653 |
| C | 0.858043 | 3.183600 | 0.676350 |
| H | 0.051346 | 2.763608 | 0.053496 |
| C | 1.622060 | 2.317748 | 1.479210 |
| C | 2.908845 | -1.394028 | 2.688660 |
| H | 2.005883 | -1.809364 | 3.162383 |
| C | 4.148429 | -2.023951 | 2.888652 |
| H | 4.211287 | -2.910285 | 3.539322 |
| C | 5.302348 | -1.535821 | 2.249006 |
| H | 6.272701 | -2.033814 | 2.400958 |
| C | 5.205028 | -0.418350 | 1.399927 |
| H | 6.097072 | -0.041558 | 0.875382 |
| C | 3.968052 | 0.221513 | 1.210465 |
| H | 3.905017 | 1.092982 | 0.542077 |

```
C      2.808356  -0.250531   1.866664
Co     -0.178155  -0.591931   0.273707
H     -0.062440  -1.798463   1.266111
```

CoP4N2+MeCN

Frequencies, energies and thermodynamic parameters:

| | |
|---|---|
| Lowest Vibrational Mode (1/cm) = | 18.2147 |
| E(RB-P86) (a.u.) = | -4693.53076362 |
| Thermal correction to Enthalpy (a.u.) = | 0.872066 |
| Thermal correction to Gibbs Free Energy (a.u.) = | 0.736174 |
| Total Entropy (cal/Kmol) = | 286.009 |

Optimised cartesian coordinates (Angstrom):

```
P     -1.170098  -0.781110   1.533749
P      1.761947  -0.959595   1.170667
P      1.766257   0.078110  -1.443234
P     -1.154683   0.480071  -1.572319
N      3.629940  -1.825960  -0.656569
N      2.853894   1.564801   0.641491
C     -1.611918  -3.505557   2.263942
H     -0.756587  -3.414531   2.948114
C     -2.235205  -4.759335   2.132880
H     -1.857077  -5.615909   2.712426
C     -3.334991  -4.917990   1.271706
H     -3.822906  -5.899839   1.170373
C     -3.802140  -3.813244   0.536977
H     -4.658052  -3.919371  -0.147144
C     -3.174610  -2.564899   0.661888
H     -3.543791  -1.714611   0.077006
C     -2.079517  -2.389128   1.537030
C     -3.775030   0.171934   2.311877
H     -4.064740  -0.871209   2.506009
C     -4.721707   1.200799   2.459390
H     -5.749019   0.954332   2.770204
C     -4.365034   2.537369   2.202003
H     -5.115057   3.337174   2.304183
C     -3.047682   2.849174   1.816695
H     -2.753183   3.891114   1.616426
C     -2.104809   1.822754   1.661314
H     -1.080249   2.058382   1.339525
C     -2.464436   0.477670   1.890800
C     -0.174196  -0.855482   3.126811
H     -0.055626   0.200078   3.445239
H     -0.750840  -1.369746   3.922694
C      1.187382  -1.504874   2.859775
H      1.117847  -2.611782   2.831342
H      1.944533  -1.241153   3.626366
C      2.850855  -2.336631   0.463740
H      3.499776  -2.779894   1.238206
H      2.156657  -3.130004   0.113827
C      5.718245  -0.903917  -1.597529
H      5.220224  -0.628591  -2.535788
C      7.085344  -0.615066  -1.473615
H      7.598592  -0.128807  -2.318461
C      7.794705  -0.927274  -0.301491
H      8.865297  -0.691750  -0.208126
C      7.095600  -1.543383   0.750220
H      7.617313  -1.801465   1.685550
```

| | | | |
|---|---|---|---|
| C | 5.728697 | -1.839418 | 0.642525 |
| H | 5.238143 | -2.314951 | 1.501420 |
| C | 4.997243 | -1.530740 | -0.540569 |
| C | 2.854583 | -1.424459 | -1.821367 |
| H | 2.154404 | -2.237977 | -2.106858 |
| H | 3.508049 | -1.231367 | -2.689149 |
| C | 3.022690 | 1.350553 | -0.780860 |
| H | 4.053641 | 1.005967 | -1.011161 |
| H | 2.845800 | 2.287836 | -1.337847 |
| C | 2.399088 | 3.973187 | 0.565592 |
| H | 3.052027 | 4.079559 | -0.313183 |
| C | 1.796372 | 5.112392 | 1.116063 |
| H | 1.983224 | 6.092235 | 0.648415 |
| C | 0.973810 | 5.020634 | 2.255497 |
| H | 0.497726 | 5.918529 | 2.676976 |
| C | 0.773854 | 3.759744 | 2.842184 |
| H | 0.122741 | 3.654954 | 3.724196 |
| C | 1.378498 | 2.613070 | 2.305076 |
| H | 1.179065 | 1.638135 | 2.766291 |
| C | 2.203140 | 2.694352 | 1.153561 |
| C | 3.058092 | 0.396877 | 1.463988 |
| H | 3.040011 | 0.672835 | 2.534199 |
| H | 4.063613 | -0.024403 | 1.253708 |
| C | 1.136083 | 0.708039 | -3.091408 |
| H | 1.193519 | 1.816809 | -3.062841 |
| H | 1.783952 | 0.356789 | -3.920317 |
| C | -0.315487 | 0.240242 | -3.238199 |
| H | -0.353441 | -0.841914 | -3.477511 |
| H | -0.858955 | 0.794791 | -4.029969 |
| C | -2.480590 | 2.832289 | -2.471440 |
| H | -3.134643 | 2.158648 | -3.045950 |
| C | -2.667301 | 4.220215 | -2.562795 |
| H | -3.466618 | 4.622524 | -3.204802 |
| C | -1.834677 | 5.092356 | -1.837174 |
| H | -1.984009 | 6.181444 | -1.903609 |
| C | -0.808803 | 4.570156 | -1.032146 |
| H | -0.146872 | 5.240351 | -0.466310 |
| C | -0.626392 | 3.179963 | -0.933884 |
| H | 0.169470 | 2.768509 | -0.294311 |
| C | -1.466467 | 2.301420 | -1.641707 |
| C | -3.050693 | -1.373114 | -2.568355 |
| H | -2.214999 | -1.838591 | -3.110645 |
| C | -4.325093 | -1.960523 | -2.637348 |
| H | -4.470357 | -2.875192 | -3.232738 |
| C | -5.407465 | -1.386955 | -1.946606 |
| H | -6.404281 | -1.851428 | -1.997268 |
| C | -5.209802 | -0.217912 | -1.188850 |
| H | -6.048838 | 0.235685 | -0.639214 |
| C | -3.937804 | 0.371592 | -1.116543 |
| H | -3.793966 | 1.280432 | -0.514028 |
| C | -2.844476 | -0.200279 | -1.808615 |
| Co | 0.156878 | -0.563761 | -0.176842 |
| N | -0.193880 | -2.276725 | -0.911177 |
| C | -0.561986 | -3.343666 | -1.239506 |
| C | -1.053850 | -4.660780 | -1.601948 |
| H | -0.273714 | -5.241784 | -2.134152 |
| H | -1.353846 | -5.209443 | -0.685282 |

```
H     -1.943389  -4.569725  -2.257905

CoP4N2+
Frequencies, energies and thermodynamic parameters:
 Lowest Vibrational Mode (1/cm) =                          5.3010
 E(RB-P86) (a.u.) =                                    -4560.83521530
 Thermal correction to Enthalpy (a.u.) =                   0.821977
 Thermal correction to Gibbs Free Energy (a.u.) =          0.694423
 Total Entropy (cal/Kmol) =                                268.460
Optimised cartesian coordinates (Angstrom):
P     -1.027709   0.558921  -1.519430
P      1.871702   0.266015  -1.313426
P      1.852317  -0.608675   1.412851
P     -1.039640  -0.118378   1.744075
N      3.790699   1.144446   0.479905
N      2.674554  -2.259721  -0.627170
C     -2.076473   3.087433  -2.342320
H     -2.706731   2.536119  -3.057757
C     -2.115523   4.490548  -2.316220
H     -2.781146   5.030817  -3.007429
C     -1.304148   5.206182  -1.414108
H     -1.337838   6.306750  -1.398301
C     -0.453806   4.514683  -0.535559
H      0.179800   5.066628   0.175448
C     -0.422694   3.110758  -0.553605
H      0.222116   2.539769   0.133315
C     -1.231416   2.388744  -1.451564
C     -3.865259   0.547496  -1.366374
H     -3.780257   1.515782  -0.853839
C     -5.124524  -0.056670  -1.515286
H     -6.018373   0.443995  -1.111555
C     -5.244423  -1.292258  -2.175309
H     -6.231900  -1.766383  -2.286015
C     -4.097822  -1.918981  -2.696475
H     -4.181568  -2.884893  -3.218298
C     -2.837842  -1.320441  -2.543090
H     -1.944145  -1.834021  -2.923687
C     -2.709320  -0.083858  -1.872708
C     -0.147995   0.389271  -3.174190
H     -0.167485  -0.685199  -3.447820
H     -0.691242   0.958885  -3.955236
C      1.288447   0.879673  -2.981029
H      1.328164   1.989412  -2.950933
H      1.974372   0.539200  -3.783095
C      3.037860   1.608711  -0.669747
H      3.702818   1.949701  -1.482098
H      2.401872   2.473456  -0.384404
C      5.805053   0.138551   1.489387
H      5.286030  -0.040615   2.439640
C      7.149418  -0.251004   1.393359
H      7.626773  -0.714996   2.270978
C      7.879545  -0.064845   0.207605
H      8.931850  -0.377818   0.136825
C      7.227579   0.528012  -0.887062
H      7.768749   0.688736  -1.832925
C      5.884328   0.924027  -0.807722
H      5.428601   1.378944  -1.696626
```

| | | | |
|---|---|---|---|
| C | 5.133968 | 0.742681 | 0.388393 |
| C | 2.991883 | 0.883385 | 1.661830 |
| H | 2.332903 | 1.754331 | 1.861374 |
| H | 3.623287 | 0.742597 | 2.556084 |
| C | 3.024427 | -1.963363 | 0.753644 |
| H | 4.074464 | -1.619685 | 0.796930 |
| H | 2.941726 | -2.864056 | 1.390844 |
| C | 0.627466 | -3.366138 | 0.212393 |
| H | 0.970677 | -3.299627 | 1.250592 |
| C | -0.555251 | -4.077482 | -0.046711 |
| H | -1.117753 | -4.494367 | 0.801392 |
| C | -1.025221 | -4.245987 | -1.356933 |
| H | -1.955654 | -4.798782 | -1.553420 |
| C | -0.290593 | -3.679390 | -2.414870 |
| H | -0.635804 | -3.797927 | -3.454016 |
| C | 0.893826 | -2.971354 | -2.174933 |
| H | 1.452107 | -2.578567 | -3.034667 |
| C | 1.406924 | -2.831067 | -0.852646 |
| C | 3.070672 | -1.206239 | -1.549074 |
| H | 3.038306 | -1.544195 | -2.600135 |
| H | 4.110955 | -0.896795 | -1.334039 |
| C | 1.233329 | -1.077004 | 3.106223 |
| H | 1.043218 | -2.170359 | 3.084315 |
| H | 2.001340 | -0.887446 | 3.883660 |
| C | -0.060999 | -0.297929 | 3.354429 |
| H | 0.162217 | 0.728311 | 3.707486 |
| H | -0.691885 | -0.777245 | 4.129183 |
| C | -3.156461 | -1.483617 | 3.096327 |
| H | -2.905050 | -0.886014 | 3.987068 |
| C | -4.235536 | -2.380157 | 3.145320 |
| H | -4.808342 | -2.496524 | 4.078693 |
| C | -4.591447 | -3.122126 | 2.001243 |
| H | -5.443074 | -3.819290 | 2.041694 |
| C | -3.861575 | -2.968741 | 0.810957 |
| H | -4.132053 | -3.541280 | -0.089167 |
| C | -2.774431 | -2.079961 | 0.765727 |
| H | -2.194369 | -1.960513 | -0.156786 |
| C | -2.417178 | -1.331417 | 1.901910 |
| C | -1.215986 | 2.626855 | 2.426023 |
| H | -0.140287 | 2.566874 | 2.654082 |
| C | -1.861262 | 3.869152 | 2.521275 |
| H | -1.290913 | 4.755613 | 2.838570 |
| C | -3.223557 | 3.985105 | 2.192019 |
| H | -3.725691 | 4.963093 | 2.251027 |
| C | -3.941196 | 2.844664 | 1.792132 |
| H | -5.010886 | 2.922787 | 1.541776 |
| C | -3.300407 | 1.596245 | 1.715408 |
| H | -3.872499 | 0.705722 | 1.418163 |
| C | -1.927896 | 1.477275 | 2.019940 |
| Co | 0.279588 | -0.225880 | 0.029809 |

FeCpCO2H

Frequencies, energies and thermodynamic parameters:

| | |
|---|---|
| Lowest Vibrational Mode (1/cm) = | 31.1495 |
| E(RB-P86) (a.u.) = | -1684.37549872 |
| Thermal correction to Enthalpy (a.u.) = | 0.117235 |
| Thermal correction to Gibbs Free Energy (a.u.) = | 0.071203 |

```
 Total Entropy (cal/Kmol) =                              96.883
Optimised cartesian coordinates (Angstrom):
Fe      0.151412  -0.000064  -0.199191
O      2.083177  -2.150414   0.090101
O      2.082453   2.150937   0.090084
C      1.308441  -1.280627  -0.013000
C      1.308045   1.280860  -0.013014
C     -1.322626  -0.713136   1.130735
C     -1.322741   0.713530   1.130300
C     -1.608023   1.159867  -0.216486
C     -1.786943  -0.000487  -1.038093
C     -1.607841  -1.160312  -0.215779
H     -2.011553  -0.000838  -2.111270
H     -1.675310   2.202894  -0.548406
H     -1.135900  -1.361037   1.995831
H     -1.674967  -2.203547  -0.547077
H     -1.136107   1.361984   1.995002
H      0.562216  -0.000156  -1.634587

FeCpCO2+MeCN
Frequencies, energies and thermodynamic parameters:
 Lowest Vibrational Mode (1/cm) =                        13.8243
 E(RB-P86) (a.u.) =                                    -1816.30822413
 Thermal correction to Enthalpy (a.u.) =                 0.160935
 Thermal correction to Gibbs Free Energy (a.u.) =        0.103637
 Total Entropy (cal/Kmol) =                              120.593
Optimised cartesian coordinates (Angstrom):
Fe      0.322537   0.098879   0.000118
O      0.240983   2.080249  -2.158049
O      0.248209   2.086850   2.152469
C      0.256014   1.301547  -1.302958
C      0.260321   1.305461   1.299780
C      2.168438  -0.567264  -0.717310
C      2.169819  -0.566094   0.715121
C      1.141468  -1.476100   1.165556
C      0.524677  -2.033552   0.001689
C      1.139261  -1.478113  -1.164249
N     -1.548626  -0.176865   0.002917
C     -2.701894  -0.353566   0.003249
C     -4.133733  -0.577907   0.002110
H     -4.358749  -1.562105  -0.455702
H     -4.519432  -0.564125   1.041227
H     -4.637757   0.217309  -0.583138
H     -0.328226  -2.723815   0.003120
H      0.883502  -1.700507   2.207256
H      2.836698   0.019576  -1.359674
H      0.879270  -1.704213  -2.205083
H      2.839343   0.021801   1.355201

FeCpCO2+
Frequencies, energies and thermodynamic parameters:
 Lowest Vibrational Mode (1/cm) =                        36.3864
 E(RB-P86) (a.u.) =                                    -1683.57234980
 Thermal correction to Enthalpy (a.u.) =                 0.109748
 Thermal correction to Gibbs Free Energy (a.u.) =        0.064193
 Total Entropy (cal/Kmol) =                              95.879
Optimised cartesian coordinates (Angstrom):
```

```
Fe     -0.107858  -0.000026  -0.406303
O     -2.033813   2.159567   0.116821
O     -2.034157  -2.159296   0.116824
C     -1.292937   1.309348  -0.132555
C     -1.293127  -1.309205  -0.132542
C      1.004994   0.719575   1.175418
C      1.005324  -0.720722   1.174754
C      1.630342  -1.167571  -0.047598
C      2.008563   0.000561  -0.780376
C      1.629936   1.167822  -0.046507
H      2.419777   0.001116  -1.799821
H      1.780791  -2.208215  -0.358540
H      0.604162   1.357048   1.973612
H      1.780006   2.208817  -0.356461
H      0.604756  -1.359101   1.972359
```

H2

```
Frequencies, energies and thermodynamic parameters:
 Lowest Vibrational Mode (1/cm) =                          4261.6436
 E(RB-P86) (a.u.) =                                       -1.17267651007
 Thermal correction to Enthalpy (a.u.) =                   0.013013
 Thermal correction to Gibbs Free Energy (a.u.) =         -0.001841
 Total Entropy (cal/Kmol) =                                31.264
Optimised cartesian coordinates (Angstrom):
H      0.000000   0.000000   0.383933
H      0.000000   0.000000  -0.383933
```

H+MeCN2

```
Frequencies, energies and thermodynamic parameters:
 Lowest Vibrational Mode (1/cm) =                          17.6651
 E(RB-P86) (a.u.) =                                       -265.758023847
 Thermal correction to Enthalpy (a.u.) =                   0.106532
 Thermal correction to Gibbs Free Energy (a.u.) =          0.060796
 Total Entropy (cal/Kmol) =                                96.258
Optimised cartesian coordinates (Angstrom):
C     -2.428295   0.000292   0.000515
N     -1.265438   0.000473   0.001148
C     -3.873489  -0.000405  -0.000725
H     -4.243418   0.899721  -0.531237
H     -4.242750  -0.909846  -0.515621
H     -4.244699   0.008473   1.043567
H      0.000087   0.000386   0.000585
N      1.265438   0.000149  -0.000038
C      2.428292  -0.000078  -0.000480
C      3.873486  -0.000204  -0.000142
H      4.243304  -0.899924   0.531130
H      4.243941  -0.010412  -1.044692
H      4.243577   0.909613   0.513491
```

H+MeCN

```
Frequencies, energies and thermodynamic parameters:
 Lowest Vibrational Mode (1/cm) =                          379.6528
 E(RB-P86) (a.u.) =                                       -133.063777356
 Thermal correction to Enthalpy (a.u.) =                   0.059331
 Thermal correction to Gibbs Free Energy (a.u.) =          0.030113
 Total Entropy (cal/Kmol) =                                61.495
Optimised cartesian coordinates (Angstrom):
```

```
C     -1.272261   0.000063   0.000074
H     -1.634556  -0.682479  -0.796376
H     -1.634102  -0.348437   0.989437
H     -1.633960   1.031282  -0.192725
C      0.160976  -0.000183  -0.000200
N      1.318402   0.000004   0.000003
H      2.341518   0.000327   0.000400
```

```
MeCN
Frequencies, energies and thermodynamic parameters:
 Lowest Vibrational Mode (1/cm) =                         379.8399
 E(RB-P86) (a.u.) =                                      -132.663448690
 Thermal correction to Enthalpy (a.u.) =                  0.048282
 Thermal correction to Gibbs Free Energy (a.u.) =         0.020680
 Total Entropy (cal/Kmol) =                               58.095
Optimised cartesian coordinates (Angstrom):
C      0.000000   0.000000   0.276029
N      0.000000   0.000000   1.448307
C      0.000000   0.000000  -1.182473
H      0.000000   1.040298  -1.566496
H      0.900924  -0.520149  -1.566496
H     -0.900924  -0.520149  -1.566496
```
